# Benchmarking framework for machine learning classification from fNIRS data

**DOI:** 10.3389/fnrgo.2023.994969

**Published:** 2023-03-03

**Authors:** Johann Benerradi, Jeremie Clos, Aleksandra Landowska, Michel F. Valstar, Max L. Wilson

**Affiliations:** School of Computer Science, University of Nottingham, Nottingham, United Kingdom

**Keywords:** fNIRS, machine learning, deep learning, open access data, neural networks, benchmarking, guidelines

## Abstract

**Background:**

While efforts to establish best practices with functional near infrared spectroscopy (fNIRS) signal processing have been published, there are still no community standards for applying machine learning to fNIRS data. Moreover, the lack of open source benchmarks and standard expectations for reporting means that published works often claim high generalisation capabilities, but with poor practices or missing details in the paper. These issues make it hard to evaluate the performance of models when it comes to choosing them for brain-computer interfaces.

**Methods:**

We present an open-source benchmarking framework, BenchNIRS, to establish a best practice machine learning methodology to evaluate models applied to fNIRS data, using five open access datasets for brain-computer interface (BCI) applications. The BenchNIRS framework, using a robust methodology with nested cross-validation, enables researchers to optimise models and evaluate them without bias. The framework also enables us to produce useful metrics and figures to detail the performance of new models for comparison. To demonstrate the utility of the framework, we present a benchmarking of six baseline models [linear discriminant analysis (LDA), support-vector machine (SVM), k-nearest neighbours (kNN), artificial neural network (ANN), convolutional neural network (CNN), and long short-term memory (LSTM)] on the five datasets and investigate the influence of different factors on the classification performance, including: number of training examples and size of the time window of each fNIRS sample used for classification. We also present results with a sliding window as opposed to simple classification of epochs, and with a personalised approach (within subject data classification) as opposed to a generalised approach (unseen subject data classification).

**Results and discussion:**

Results show that the performance is typically lower than the scores often reported in literature, and without great differences between models, highlighting that predicting unseen data remains a difficult task. Our benchmarking framework provides future authors, who are achieving significant high classification scores, with a tool to demonstrate the advances in a comparable way. To complement our framework, we contribute a set of recommendations for methodology decisions and writing papers, when applying machine learning to fNIRS data.

## 1. Introduction

Many research fields, including computer vision and natural language processing, benefit from strong standards, with state-of-the-art models, and established ways to benchmark machine learning on common datasets (LeCun et al., 1998; Krizhevsky and Hinto, 2009; Maas et al., 2011). For relatively new areas of application, like the classification of fNIRS data however, our community still lacks clear standards and approaches to compare and recognise significant advances in performance. This lack of standards creates a large discrepancy in how machine learning is applied to fNIRS, and how the methodology and results are reported in fNIRS papers, and this makes it hard to draw clear conclusions as to whether some approaches are really better than others. Notably, fNIRS machine learning papers sometimes fall foul of common mistakes, and the way that methods and results are presented are often missing critical information that would make them reproducible. These problems are exacerbated by the fact that the field lacks commonly recognised open access datasets for machine learning benchmarking, even though this journey is going in the right direction with the more frequent publication of open access datasets and a will to gather them in a single place.^1^ Moreover, the lack of code sharing practices, which would enable inspection by others and improve reproducibility, is another issue that ultimately slows the progress of our field.

The same way the fNIRS community is going toward more established practices for signal processing (Pinti et al., 2018b; Santosa et al., 2020) and reporting (Yücel et al., 2021), we aim in this research to provide a community resource specifically for machine learning in the context of fNIRS BCI applications. Our work enables researchers to: (1) reuse the implementation of a robust machine learning framework methodology on common open access fNIRS datasets in an open source code repository, (2) share the implementation of fNIRS machine learning approaches such that they can be inspected and validated by others, (3) apply new machine learning approaches easily on multiple common open access fNIRS datasets such that they can be compared to baseline implementations as well as recent contributions, and (4) contribute to a community best practice checklist of expectations for both decisions made during implementation and analysis, and for reporting detail in papers.

Since in-depth comparisons of signal processing pipelines have already been conducted in the literature (Pinti et al., 2018b; Santosa et al., 2020), this paper will use a signal processing pipeline based on those recommended best practices for all the experiments and focus on comparing various machine learning algorithms with a robust methodology. More specifically, we describe the implementation of a range of baseline machine learning algorithms on a specific set of public datasets in Section 2, and present the results of such a multi-algorithm multi-dataset benchmarking comparison in Section 3. Finally in Section 4 we present a recommended checklist for researchers that are implementing machine learning approaches for classification from fNIRS data (Section 4.7) and the details of the Python framework developed to perform multi-dataset comparison with a robust machine learning methodology (Section 4.8).

Further, we consider this work as a call to action, toward helping the community establish, from the variety of unstandardised approaches that have been published so far, consolidated best practices for identifying advances in our community. We list our initial recommended practices in this paper, but we invite community members to contribute to a growing working document of best practices on our repository.^2^

### 1.1. fNIRS and brain-computer interfaces

Even though electroencephalography (EEG) is the most popular brain imaging technique for BCIs, research into continuous wave fNIRS is increasing due to its relative tolerance to user motion (Nishiyori, 2016). fNIRS is based on the absorption properties of hemoglobin in the near infrared spectrum (Jobsis, 1977) and enables us to measure relative changes in both oxyhemoglobin (HbO) and deoxyhemoglobin (HbR) (Delpy et al., 1988). It is characterised by a lower temporal resolution but is capable of higher spacial resolution than EEG (Nishiyori, 2016), and the frequencies of interest with fNIRS (under 1 Hz) are usually lower than the ones studied with EEG (Clerc et al., 2016; Rahman et al., 2019). fNIRS still faces challenges to be used reliably in real life conditions, but more and more lab settings experiments are working toward this goal (Pinti et al., 2018a), with some studies focusing for instance on walking (Vitorio et al., 2017) and climbing (Carius et al., 2020). fNIRS is currently mostly used for passive (rather than active) BCIs (Zander and Kothe, 2011; Zephaniah and Kim, 2014) due to the 1–2 s delay in cerebral blood flow and a peak response 4–6 s after a stimulus (Buxton et al., 2004).

A lot of tasks have been used in lab settings in order to advance BCI research. The first category falls under the *active BCI* category, where the user actively attempts to control an application through purposeful thought (Clerc et al., 2016). Researchers often use motor tasks for this purpose, where finger tapping is most commonly used in fNIRS research (Sitaram et al., 2007; Cui et al., 2010). Research can also involve motor imagery (Pfurtscheller and Neuper, 2001), which consists of imagining a movement without actually performing it. Indeed, motor imagery has been shown to elicit similar brain activity to motor execution (Miller et al., 2010).

For *passive BCIs*, which are not used to voluntarily control an application, fNIRS data is used to monitor and classify a user's mental state (Clerc et al., 2016) while they perform a task. A range of different mental workload tasks are often used to train such passive BCIs (Maior et al., 2014; Benerradi et al., 2019). One of the most popular is the n-back task, which involves remembering the recurrence of regularly presented stimuli (Wang et al., 2015; Aghajani et al., 2017; Le et al., 2018). A second task used to elicit mental workload is the word generation task, where subjects are asked to give as many word starting by a designated letter as possible (Faress and Chau, 2013; Hong et al., 2018). Finally, mental arithmetic tasks are often used, in which subjects are asked to solve simple mathematics operations such as additions, subtractions, multiplications, and divisions (Hong et al., 2018; Yoo et al., 2018).

### 1.2. Machine learning for fNIRS brain-computer interfaces

Many standard machine learning approaches have been used to classify fNIRS data in the context of different tasks and applications. For instance, Herff et al. (2013) used LDA to classify mental tasks describing results of 71, 70, and 62% accuracy on mental arithmetic, word generation and mental rotation tasks respectively against rest. Hong et al. (2015) used LDA to classify between mental arithmetic, left- and right-hand motor imagery and obtained and average classification accuracy of 75.6%. Nazeer et al. (2020) also used LDA with features extracted using vector-based phase analysis on finger tapping tasks presenting classification accuracies of 98.7 and 85.4% with two classes (left-hand, right-hand) and three classes (left-hand, right-hand, rest), respectively. Shin et al. (2016a) also used SVM for classifying mental arithmetic against baseline and obtained performances around 77% with eyes opened and around 75% with eyes closed. Other standard machine learning models such as kNN have been also used, for example, to classify three different mental workload levels on n-back tasks and reached accuracies up to 52.08% (Kesedžić et al., 2020).

Deep learning has also been used extensively with fNIRS data to classify activity during tasks. For example, Chan et al. (2012) used an ANN and reported an accuracy of 63.0% for classification of mental signing against rest. Trakoolwilaiwan et al. (2017) compared an ANN and a CNN to classify between left-, right-hand finger tapping and rest and report accuracies of 89.35 and 92.68%, respectively. Yoo et al. (2018) used a LSTM model for classification between mental arithmetic, mental counting, and puzzle solving, and report accuracies up to 83.3%.

The classification performances reported are extremely high, and would suggest that this research is ready for technology transfer by industry, however our findings suggest otherwise.

### 1.3. Limitations of current literature

Recent work has produced strong examples for recommending best practices for processing of fNIRS data (Pinti et al., 2018b), and considerations regarding the reporting of works with fNIRS (Yücel et al., 2021). In this trend, we highlight issues specific to machine learning classification with fNIRS data.

Machine learning, while popular, often suffers from flaws in many existing publications across various domains of applications (Kapoor and Narayanan, 2022), including in the domain of BCIs (Nakanishi et al., 2020). In reviewing the literature of machine learning applied to fNIRS, it is common to see limitations that can be categorised into two types.^3^ The first type regards the methodology, including potential mistakes, flaws, and lack of rigor. Common issues with published research include:

- not taking into consideration the experimental design when selecting instances to classify, this includes for example using resting periods used for return to baseline the same way as intentionally designed control baseline tasks;
- randomly selecting hyperparameters without justification and not performing hyperparameter tuning;
- optimising the model's hyperparameters using the test set (also called overfitting to the hyperparameters);
- testing classifiers with data already seen during training and neglecting the potential overlap between the different sets (training, validation, test), this includes for example issues related to sliding windows with overlapping;
- not using cross-validation or permutation testing to validate results;
- not performing a statistical analysis to compare results;
- not handling class imbalance.

This first type of limitations is however difficult to highlight with certainty in most cases, this being related to the second type of limitations which is the reporting of works using machine learning with fNIRS. This makes reproducibility of previous works often impossible, which is even more problematic when the data and/or the code are not available. Those limitations include:

- not explaining what data is used as input of the classifier;
- not providing enough details regarding the machine learning models, including for example the hyperparameters or the architecture;
- not describing the split between training, validation and test sets, and how many input examples are used (in this manuscript we call *example* an instance used as one input of a machine learning model);
- not referring results to the number of classes or chance level.

All those issues often make it hard to be confident when writing related work sections in machine learning papers with fNIRS, especially when it comes to reporting the state-of-the-art results because they are not reproducible in most cases.

### 1.4. Research questions

Overall, our three key research questions are:

- *RQ1*: How can we make the comparison of machine learning approaches for task classification from fNIRS data more rigorous and robust?
- *RQ2*: What are the benchmarkings of popular machine learning models on various tasks from open access fNIRS datasets?
- *RQ3*: Across these benchmarks, what factors influence the machine learning classification accuracy?

Further, as we delve into the specifics of what influences classification accuracy (RQ3), we ask:

- *RQ3a*: What is the influence of the number of examples used for training machine learning models on classification accuracy?
- *RQ3b*: What is the influence of the time window length of inputs on classification accuracy?
- *RQ3c*: What is the influence of using a sliding window on classification accuracy as opposed to using epochs starting at the beginning of each task?
- *RQ3d*: What is the influence of using a personalised approach (classification of data within subjects) on classification accuracy as opposed to a generalised approach (classification of data from unseen subjects)?

## 2. Methods

### 2.1. Data

Multiple datasets were used for this comparison of machine learning methods with fNIRS. The focus was put here on cognitive tasks as it is an important domain of application for fNIRS, but one dataset with a motor task was also used for comparison purposes. They were also chosen based on characteristic of having at least a sampling frequency of 10 Hz as recommended by Yücel et al. (2021) so that optode-scalp coupling can be checked. All the datasets used are openly accessible and have been produced as part of previous studies by researchers of the fNIRS community. Appropriate ethical approvals were attained as stated in the datasets' dedicated papers, and participants gave written informed consent.

#### 2.1.1. n-back dataset collected by Herff et al. in 2014

This dataset consists of n-back tasks performed by 10 healthy participants. The experiment consisted, for each participant, of 10 epochs of each 1-back, 2-back, and 3-back; each epoch containing 3 ± 1 targets. Each epoch consisted of 5 s of instruction, 44 s of n-back with a letter every 2 s displayed for 500 ms, and a 15 s rest period. The data was recorded with an OxyMon Mark III from Artinis Medical Systems, with wavelengths of 765 and 856 nm and a sampling rate of 25 Hz. It is composed of four sources and four detectors on the prefrontal cortex (PFC), resulting in eight channels of HbO and eight channels of HbR, with a source-detector distance of 35 mm. More details can be found in Herff et al. (2014). This dataset has been used for classification between 1-back, 2-back, and 3-back.

#### 2.1.2. n-back dataset collected by Shin et al. in 2018

This dataset consists of n-back tasks performed by 26 healthy participants. The experiment consisted, for each participant, of nine epochs (divided into three sessions) of each 0-back, 2-back, and 3-back. Each epoch consisted of 2 s of instructions, 40 s of task and 20 s of rest period. A random digit was given every 2 s displayed for 0.5 s and the targets appeared with a 30% chance. The data was recorded with a NIRScout from NIRx Medical Technologies, with wavelengths of 760 and 850 nm and a sampling rate of 10 Hz. It is composed of 16 sources and 16 detectors on the PFC, resulting in 36 channels of HbO and 36 channels of HbR, with a source-detector distance of 30 mm. More details can be found in Shin et al. (2018). This dataset has been used for classification between 0-back, 2-back, and 3-back.

#### 2.1.3. Word generation dataset collected by Shin et al. in 2018

This dataset consists in word generation tasks performed by the same 26 healthy participants as the previous dataset. The experiment consisted, for each participant, of 30 epochs (divided into three sessions) of each word generation and baseline task. Each epoch consisted of a 2 s instruction showing an initial single letter for word generation or the fixation cross for baseline, a 10 s task period with a fixation cross, and a 13–15 s rest period also with a fixation cross. The hardware settings were the same as the previous dataset. More details can be found in Shin et al. (2018). This dataset has been used for classification between baseline task and word generation.

#### 2.1.4. Mental arithmetic dataset collected by Shin et al. in 2016

This dataset consists of mental arithmetic tasks performed by 29 healthy participants. The experiment consisted, for each participant, of 30 epochs (divided into three sessions) of each mental arithmetic and baseline task. Each epoch displayed the subtraction for 2 s, had a 10 s task period with a fixation cross, and a 15–17 s rest period also with a fixation cross. The data was recorded with a NIRScout from NIRx Medical Technologies, with a sampling rate of 10 Hz. It is composed of 14 sources and 16 detectors on the PFC, resulting in 36 channels at 760 nm and 36 channels at 850 nm, with a source-detector distance of 30 mm. More details can be found in Shin et al. (2016b). This dataset has been used for classification between baseline task and mental arithmetic.

#### 2.1.5. Motor execution dataset collected by Bak et al. in 2019

This dataset consists of finger and foot tapping tasks performed by 30 healthy participants. The experiment consisted, for each participant, of 25 epochs of each right-hand finger tapping, left-hand finger tapping and foot tapping. Each epoch contained a 2 s introduction, 10 s of actual task and a 17–19 s rest period. The finger tapping was performed at 2 Hz and the foot tapping at 1 Hz. The data was recorded with a LIGHTNIRS from Shimadzu, with a sampling rate of 13.3 Hz. It is composed of eight sources and eight detectors around the motor cortex, resulting in 20 channels of HbO and 20 channels of HbR, with a source-detector distance of 30 mm. More details can be found in Bak et al. (2019). This dataset has been used for classification between right-hand finger tapping, left-hand finger tapping, and foot tapping.

### 2.2. Signal processing and data cleansing

Datasets from Herff et al. (2014), Shin et al. (2018), and Bak et al. (2019) provided HbO and HbR concentration change data while the dataset from Shin et al. (2016b) provided light intensity data. This is why data from Shin et al. (2016b) was first converted into optical density changes, relative to the average on the whole measurements for each channel. Then the modified Beer-Lambert law (MBLL) (Delpy et al., 1988) was applied to obtain changes in HbO and HbR. The Wray et al. (1988) molar extinction coefficient table was used and the differential pathlength factor (DPF)s set to 6.0, as those are the most common in the literature and fNIRS softwares. As per Shin et al. (2016b), the source-detector distances used were 3 cm. This preprocessing was performed with the NIRSimple library^4^ (version 0.1.2) created for fNIRS preprocessing in Python and giving control on many parameters including the choice between different molar extinction coefficient tables from the literature.

The rest of the signal processing has been performed with MNE-Python version 0.23.4 (Gramfort et al., 2013) with methods as follow. From HbO and HbR changes the data was first corrected with temporal derivative distribution repair (TDDR) (Fishburn et al., 2019) to remove motion artifacts and then bandpass filtered with an infinite impulse response (IIR) Butterworth filter of order 4. The band-pass edges of 0.01 and 0.5 Hz were used to remove noise related to heart beat and slow drifts (Naseer and Hong, 2015), without clashing with the experimental design of the different datasets used in our work (task durations ranging from 10 to 44 s resulting in task frequencies from 1/44 = 0.02 to 1/10 = 0.1 Hz). The channels were averaged by region of interest (Poldrack, 2007; Naseer and Hong, 2015) to end up with a left-side average and right-side average for each HbO and HbR in the appropriate brain area depending on the task:

- mental workload tasks such as n-back, mental arithmetic and word generation have been shown to elicit brain activity in the PFC so the region of interest side averages were performed in that area for the mental workload task datasets (Naseer and Hong, 2015; Friedman and Robbins, 2021);
- motor execution have been shown to elicit brain activity in the motor cortex so the region of interest side averages were performed in that area for the motor execution task dataset (Naseer and Hong, 2015; Bhattacharjee et al., 2021).

This resulted in a total of four regions of interest for each dataset (a detail of the regions of interest can be found in the Supplementary material). This region of interest averaging was made to have the same resulting number of channels for each dataset in the comparison, since different fNIRS devices with different number of optodes were used. The measurements were then epoched thanks to the onset triggers according to each dataset description, and a baseline correction was performed such that the average concentration change on the baseline prior to each task is null for each region of interest of each type. The baseline duration used was the instruction segments just prior to each task. For comparison purposes between the different datasets, the shortest duration of instruction of 2 s (from Shin et al., 2018; Bak et al., 2019) was used for every dataset, meaning that longer durations would be cropped down to 2 s. The epochs were down-sampled to 10 Hz so that every dataset ends up with the same sampling frequency and to reduce computing demand for the machine learning execution. Finally, every epoch of every dataset was cropped down to the shortest epoch duration available of 10 s from the onset triggers (Shin et al., 2016b, 2018; Bak et al., 2019) for easier comparison between datasets.

Finally the data in M was converted into μM, and the baseline was cropped such that only the task segments are used as inputs for following analysis. Table 1 summarises the size of each dataset. In the end, the shape of each example is 4 × 100, representing 2 channels of HbO and 2 channels of HbR by 100 time points (10 s of 10 Hz signals).

**Table 1:** Information about the datasets.

**Dataset**	**Classes**	**No. of participants**	**No. of examples per class**
Herff et al. (2014)	1-back; 2-back; 3-back	10	100
n-back			
Shin et al. (2018)	0-back; 2-back; 3-back	26	234
n-back			
Shin et al. (2018)	Baseline; word generation	26	780
word generation			
Shin et al. (2016b)	Baseline; mental arithmetic	29	870
mental arithmetic			
Bak et al. (2019)	Right hand; left hand; foot	30	750
motor execution			

### 2.3. Feature extraction

Temporal features have been extracted and used as input of four of the six models tested here: the LDA, SVM, kNN, and ANN. This was not done for the CNN to let it extract features from raw data and the LSTM to let it learn temporal dependencies from the raw data. The features extracted here are three of the most popular in the fNIRS literature (Naseer and Hong, 2015):

- the mean for each region of interest of each type across time;
- the standard deviation for each region of interest of each type across time;
- the slope of the linear regression for each region of interest of each type across time.

### 2.4. Machine learning models

Six supervised machine learning models were compared on all the datasets, this includes three standard machine learning models and three deep learning models.

#### 2.4.1. Standard machine learning

Firstly, three standard machine learning models are implemented:

- LDA (Cohen et al., 2014) classifiers learn a linear decision surface to split the data into the different classes. They have the advantage of not having any hyperparameter to tune and a low computational cost. The LDA model implemented in this work uses the features extracted from the signals as described above.
- SVM (Hearst et al., 1998) classifiers or support-vector classifier (SVC)s aim to find a hyperplane able to separate the data with maximal margin with respect to data points of each class. A SVC with a linear kernel or linear SVC uses a linear decision surface similarly to the LDA with the difference of it being fitted with margin maximisation. It uses a regularisation hyperparameter that needs to be tuned. The SVC implemented in this work also uses the features extracted from the signals. The regularisation parameter was optimised following the hyperparameter tuning procedure described bellow. The maximum number of iterations was set to 250,000 in order to guarantee convergence.
- kNN (Altman, 1992) is a non-parametric classification algorithm using the closest k points from the training data in order to make a prediction on the class. Here, we use a majority vote with those labeled k points. The kNN classifier implemented here also uses features extracted from the signals, and the algorithm uses a uniform weighting of the k nearest neighbours meaning that each point is weighted equally in the voting. The number of neighbours k is a hyperparameter of the algorithm that is tuned according to the same procedure as the other models described below.

Scikit-learn (Pedregosa et al., 2011) version 0.24.2 was used for the implementation of those standard machine learning models.

#### 2.4.2. Deep learning

Secondly, three deep-learning models are implemented:

- ANNs (McCulloch and Pitts, 1943) are the simplest type of neural network. Neural networks are composed of units called artificial neurons arranged into layers, which outputs are computed by a non-linear function of the weighted sum of it inputs from the previous layer. This process happens until the last layer, where a probability distribution for the classes is computed, enabling to get a prediction. The ANN implemented here uses features extracted from the signals as well. Hence, the input layer is composed of 12 neurons, followed by two fully connected layers of respectively 8 and 4 neurons, finished by an output layer of 2 or 3 neurons depending on the number of classes for the dataset. It uses ReLu as the activation function for each layer, Adam as the optimiser and a cross-entropy loss. The learning rate and mini-batch size were optimised following the hyperparameter tuning procedure described bellow.
- CNNs (LeCun et al., 1989) are an extensions of neural networks to which convolutional and pooling layers have been added. They use kernels sliding along the input dimensions so that it is transformed in a space invariant way. A CNN is typically composed of convolutional layers followed by standard neural network layers. The CNN implemented here uses the signal processed data without any feature extraction prior to that. It is composed of two one-dimensional convolutional layers across the time axis: the first one with four input channels and four output channels, a kernel size of 10 (one dimension kernel) and a stride of 2; the second one with four input channels and four output channels, a kernel size of 5 (one dimension kernel) and a stride of 2. Each convolutional layer is followed by a one-dimensional max pooling across the time axis with a kernel size of 2. Those convolutions and max poolings are followed by two fully connected layers of 20 and 10 neurons, respectively, followed to finish with by an output layer of 2 or 3 neurons depending on the number of classes for the dataset. It uses ReLu as the activation function for each layer, Adam as the optimiser and a cross-entropy loss. The learning rate and mini-batch size were optimised following the procedure described bellow.
- LSTM neural networks (Hochreiter and Schmidhuber, 1997) belong to the family of recurent neural network (RNN)s. RNNs (Rumelhart et al., 1986) can be seen as simple neural networks allowing new inputs of a sequence to be treated in the context of previous inputs of that sequence. LSTMs are an extension of that using a memory cell in order to learn longer-term dependencies. They are useful compared to RNNs as they allow to overcome the vanishing gradient problem. The LSTM implemented here uses the signal processed data without feature extraction as well. It uses one LSTM recurring unit with an input size of 80 (each input being arranged as a sequence of five elements of 2 s of 10 Hz data on four channels) and a hidden size of 36. It is then followed by a fully connected layer of 16 neurons, followed to finish with by an output layer of 2 or 3 neurons depending on the number of classes of the dataset. The model uses ReLu as the activation function for each layer except the LSTM unit using tanh, Adam as the optimiser and a cross-entropy loss function. Again, the learning rate and the mini-batch size were optimised following the procedure described bellow.

PyTorch (Paszke et al., 2019) version 1.5.1 was used for the implementation of those deep learning models and the models were run on the CPU as GPUs did not provide much time advantage. The CPU was an Intel Xeon E5 v3 processor.

### 2.5. Procedure and metrics

Each dataset was analysed separately, and a nested cross-validation approach was used. Statistical tests were then used to test various assumptions as described, the threshold of significance was set at 5% for all of them. All the statistical tests were performed with SciPy (Virtanen et al., 2020) version 1.8.1.

#### 2.5.1. Generalised approach

The outer cross-validation consisted in a group five-fold cross-validation to leave the test set out, such that a same subject's data cannot end up in both the training and validation set and the test set. This outer cross-validation was used to evaluate the different machine learning models for each dataset. This was made so that the results reflect the performance of a classifier of data from unseen subjects. The inner cross-validation consisted in another group three-fold cross-validation to separate training and validation set. This inner cross-validation was used for hyperparameter optimisation, choosing hyperparameters based on the accuracy on the validation set (accuracy was chosen since the classes of every dataset were perfectly balanced).

The hyperparameters optimised for the standard machine learning approaches were the regularisation parameter for the SVC and k the number of nearest neighbours for the kNN. For the deep learning approaches, the learning rate and the mini-batch size were optimised, as those parameters are known to influence models the most (Bengio, 2012). For every deep learning model, the Adam optimiser was used due to its good and reliable performance across many deep learning problems (Schmidt et al., 2020). It is an optimiser based on adaptive estimates of lower-order moments (Kingma and Ba, 2014).

In addition to that, early stopping was also performed for the deep learning model's number of epochs to avoid overfitting. This early stopping was done after the best hyperparameters were found and the model was retrained on the whole training and validation set except 20% randomly left out to perform this early stopping. This consisted in stopping deep learning training before the maximum number of epoch of 100 if the loss on the 20% left out increased (non-strictly) for five consecutive epochs.

The optimisation was done within the following ranges using grid search following common machine learning practice (Pedregosa et al., 2011; Bengio, 2012):

- the regularisation parameter's values tested were 0.001, 0.01, 0.1, and 1;
- the values of k (number of nearest neighbours) tested were the integers from 1 to 9;
- the learning rate's values tested were 1 × 10^−5^, 1 × 10^−4^, 1 × 10^−3^, 1 × 10^−2^, 1 × 10^−1^;
- the min-batch sizes tested were 4, 8, 16, 32, and 64.

Overall, the parts of the method that could be affected by the random seed were:

- the shuffling of the training and validation set (this has an influence on the SVC and the deep learning models);
- the selection of the 20% validation set used for early stopping;
- the weight initialisation of the deep learning models.

Regarding the results, the accuracies on each of the five outer folds were averaged to determine the overall accuracy for each model of each dataset. This metric was used for reference rather than others such as F1 score because of its simplicity and the perfect class balance of each dataset (no epoch rejection was performed).

For each model and each dataset, a one-tailed *t*-test was used (using the accuracy values on each outer fold) to determine whether its accuracy was greater than chance level, if the distribution of the accuracies of the model on the outer folds followed a normal distribution as tested with a Shapiro test; otherwise a one-tailed Wilcoxon test was used.

Next, a statistical analysis was run with the accuracy values on each outer fold to compare models to each other within each dataset. For this purpose a one-way analysis of variance (ANOVA) test was performed if the normality and the homoscedasticity are not excluded with Shapiro tests and a Bartlett test, respectively, otherwise a non-parametric Kruskal–Wallis test was used. If an effect of the model was identified, one-tailed paired t-tests with Bonferroni correction were used to compare each model against each other.

In addition to those results, a confusion matrix was produced for each model of each dataset, comparing the predictions made across all the outer folds against the true class labels (those can be found in the Supplementary material).

#### 2.5.2. Influence of the number of training examples on the generalised approach

In addition to comparing the different models, the influence of the training set size was also studied. For that purpose, after leaving out the test set, a proportion of the training and validation set was discarded. This way we studied variations from 0 to 50% discarded of the training data by stride of 10% for every dataset. The same procedure was then applied in terms of validation and hyperparameter search.

The correlation between the training set proportion used and the accuracy was studied with a Pearson's correlation test for each model of each dataset if the assumption of normality was verified as per a Shapiro test, otherwise a Spearman test was used.

#### 2.5.3. Influence of the time window length on the generalised approach

The influence of the time window length was also studied. We compared here epochs of 2, 3, 4, 5, 6, 7, 8, 9, and 10 s from the onset trigger marking the start of each task. Again, the same procedure of validation and hyperparameter search was used.

The same way as before, correlations between the window length and the accuracy were studied with a Pearson's correlation test for each model of each dataset if the assumption of normality was verified as per a Shapiro test, otherwise a Spearman's correlation test was used.

This approach enabled the comparison of the four models using feature extraction: LDA, SVC, kNN, and ANN. This is because comparing the models using the data without feature extraction would have required to change the architecture of those models for each window length which would have added too much variables in the comparison.

#### 2.5.4. Generalised approach with sliding window

Finally, the same procedure as the generalised approach was used but with a 2 s sliding window on the 10 s epochs instead of the 10 s at once. No overlapping was used between the different time windows, and a prediction was done for each time window.

This approach enabled the comparison of the four models using feature extraction: LDA, SVC, kNN, and ANN. This is because comparing the models using the data without feature extraction would have required to change the architecture of those models compared to the initial approach.

#### 2.5.5. Personalised approach

The same procedure as the generalised approach was used but with each participant of each dataset individually. The only difference being that the outer and inner cross-validations consisted in stratified five- and three-fold cross-validations, respectively instead of group k-fold, such that the class distribution remained balanced in the training, validation, and test sets.

## 3. Results

### 3.1. Generalised approach

The models are first compared to each other with the maximum number of training examples available and the maximum time window length of 10 s, which took 29 h and 7 min to run with the configuration described in Section 2.

**Figure 1:**
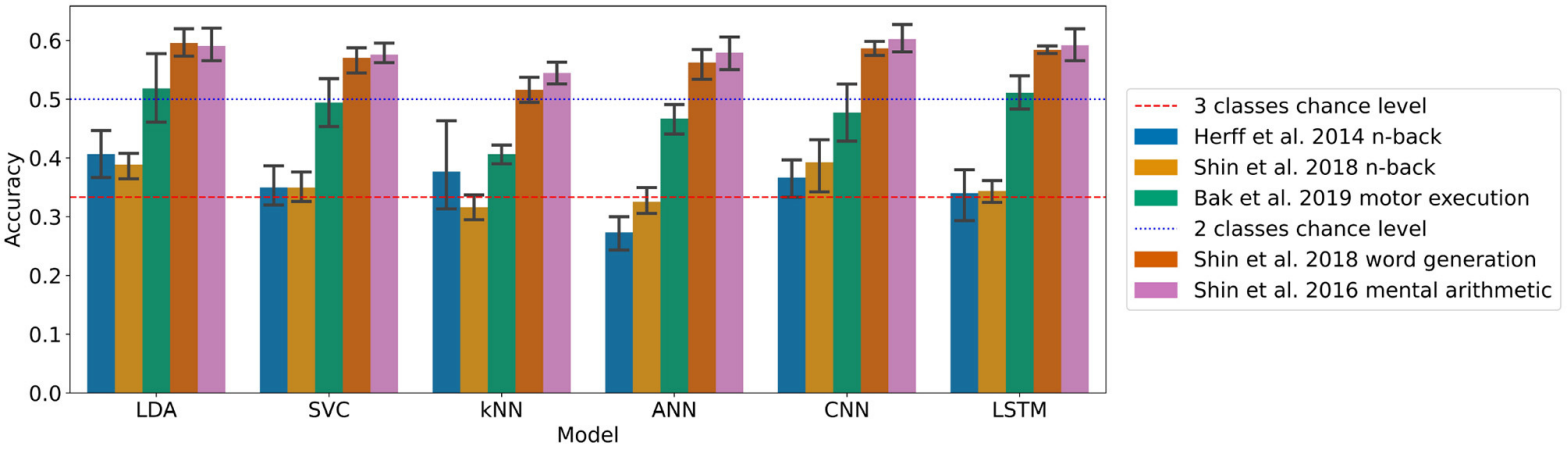
Accuracies of the models on each dataset with the generalised approach with (95% confidence intervals related to the variability on the five-fold outer cross-validation are displayed).

The results for each dataset can be found in Figure 1 and Table 2.

**Table 2:** Accuracies of the models on each dataset with the generalised approach.

**Dataset**	**Chance level**	**LDA**	**SVC**	**kNN**	**ANN**	**CNN**	**LSTM**
Herff et al. (2014)	0.333	0.407^*^	0.350	0.377	0.273	0.367	0.340
n-back		(0.049)	(0.038)	(0.083)	(0.033)	(0.038)	(0.049)
Shin et al. (2018)	0.333	0.389^*^	0.350	0.316	0.325	0.393^*^	0.344
n-back		(0.026)	(0.029)	(0.026)	(0.026)	(0.049)	(0.021)
Shin et al. (2018)	0.500	0.596^*^	0.570^*^	0.516	0.562^*^	0.587^*^	0.584^*^
word generation		(0.026)	(0.026)	(0.027)	(0.028)	(0.013)	(0.008)
Shin et al. (2016b)	0.500	0.591^*^	0.576^*^	0.545^*^	0.579^*^	0.602^*^	0.592^*^
mental arithmetic		(0.035)	(0.021)	(0.022)	(0.033)	(0.026)	(0.030)
Bak et al. (2019)	0.333	0.518^*^	0.494^*^	0.407^*^	0.467^*^	0.477^*^	0.511^*^
motor execution		(0.068)	(0.048)	(0.019)	(0.030)	(0.059)	(0.033)

Fields marked with an asterisk indicate an accuracy significantly greater than chance level at a 5% threshold, the standard deviation on the five-fold outer cross-validation is in parenthesis.

On the n-back tasks from Herff et al. (2014) and Shin et al. (2018) the accuracy was found significantly higher than chance level (33.3%) with the LDA with *p*-values of 0.020 and 0.006, respectively, reaching with three classes 40.7 and 38.9%, respectively. For the Shin et al. (2018) dataset of n-back tasks the CNN accuracy of 39.3% was also found significantly higher than chance level with a *p*-value of 0.037.

For the Shin et al. (2018) dataset of word generation tasks, significant differences compared to chance level (50%) were found for the LDA, SVC, ANN, CNN, and LSTM with *p*-values of 0.001, 0.031, 0.005, <0.001, and <0.001, respectively. For those models, the accuracy ranges from 57.0 to 59.6% for standard machine learning and from 56.2 to 58.7% for deep learning. A Wilcoxon test was used to test the significance on the SVC due to the non-normality of the distribution as measured with a Shapiro test.

For the Shin et al. (2016b) dataset of mental arithmetic tasks, significant differences compared to chance level (50%) were found for all the models with p-values ranging from 0.001 to 0.004. A Wilcoxon test was used here to test the significance on the kNN due to the non-normality of the distribution as measured with a Shapiro test. The accuracies range from 54.5 to 59.1% for the machine learning models and from 57.9 to 60.2% for the deep learning models.

Finally, for the Bak et al. (2019) dataset of motor execution tasks, significant differences compared to chance level (33.3%) were also found for all the models implemented with *p*-values up to 0.004. The accuracies range from 40.7 to 51.8% for the machine learning models and from 46.7 to 51.1% for the deep learning models.

A statistical influence of the model on the accuracy was found for each dataset except the Shin et al. (2016b) dataset of mental arithmetic tasks (Kruskal–Wallis tests were used for the Shin et al. (2018) dataset of word generation and the Shin et al. (2016b) dataset of mental arithmetic because of non-normality). More specifically with pairwise *t*-tests, on the Shin et al. (2018) dataset of n-back tasks, the accuracy of the LDA was found significantly greater than the accuracy of the kNN. On the Shin et al. (2018) dataset of word generation tasks, the LDA and CNN accuracies were found significantly greater than the kNN. Finally on the Bak et al. (2019) dataset of motor execution tasks, the LSTM accuracy was found significantly greater than the kNN and ANN accuracies.

A detail of the hyperparameters selected with grid search for each iteration of the outer cross-validation can be found in the Supplementary material, as well as the results of the statistical tests.

### 3.2. Influence of the number of training examples on the generalised approach

The correlation between the number of training examples and the classification accuracy is shown, for each dataset and each model, in Table 3. This took 93 h and 15 min to run.

**Table 3:** Correlation coefficients of the relationship between accuracy and number of training examples.

**Dataset**	**LDA**	**SVC**	**kNN**	**ANN**	**CNN**	**LSTM**
Herff et al. (2014)	0.111	0.181	−0.378^*^	−0.340	0.043	−0.342
n-back					
Shin et al. (2018)	0.087	0.026	0.083	−0.333	0.317	0.160
n-back					
Shin et al. (2018)	0.148	−0.206	−0.080	−0.035	0.243	0.007
word generation					
Shin et al. (2016b)	−0.102	−0.120	0.128	−0.268	−0.123	−0.008
mental arithmetic					
Bak et al. (2019)	0.120	0.059	0.265	0.143	0.390^*^	0.187
motor execution					

Fields marked with an asterisk indicate a significant correlation at a 5% threshold.

Only two significant correlations were found with a threshold of 5%. The kNN on the Herff et al. dataset of n-back tasks was negatively influenced by a increase in training examples with a p-value of 0.039 and a correlation coefficient of −0.378. The other one was with the CNN on the Bak et al. (2019) dataset of motor execution tasks where the accuracy was positively influenced by the number of training examples with a *p*-value of 0.033 and a correlation coefficient of 0.390.

### 3.3. Influence of the time window length on the generalised approach

**Figure 2:**
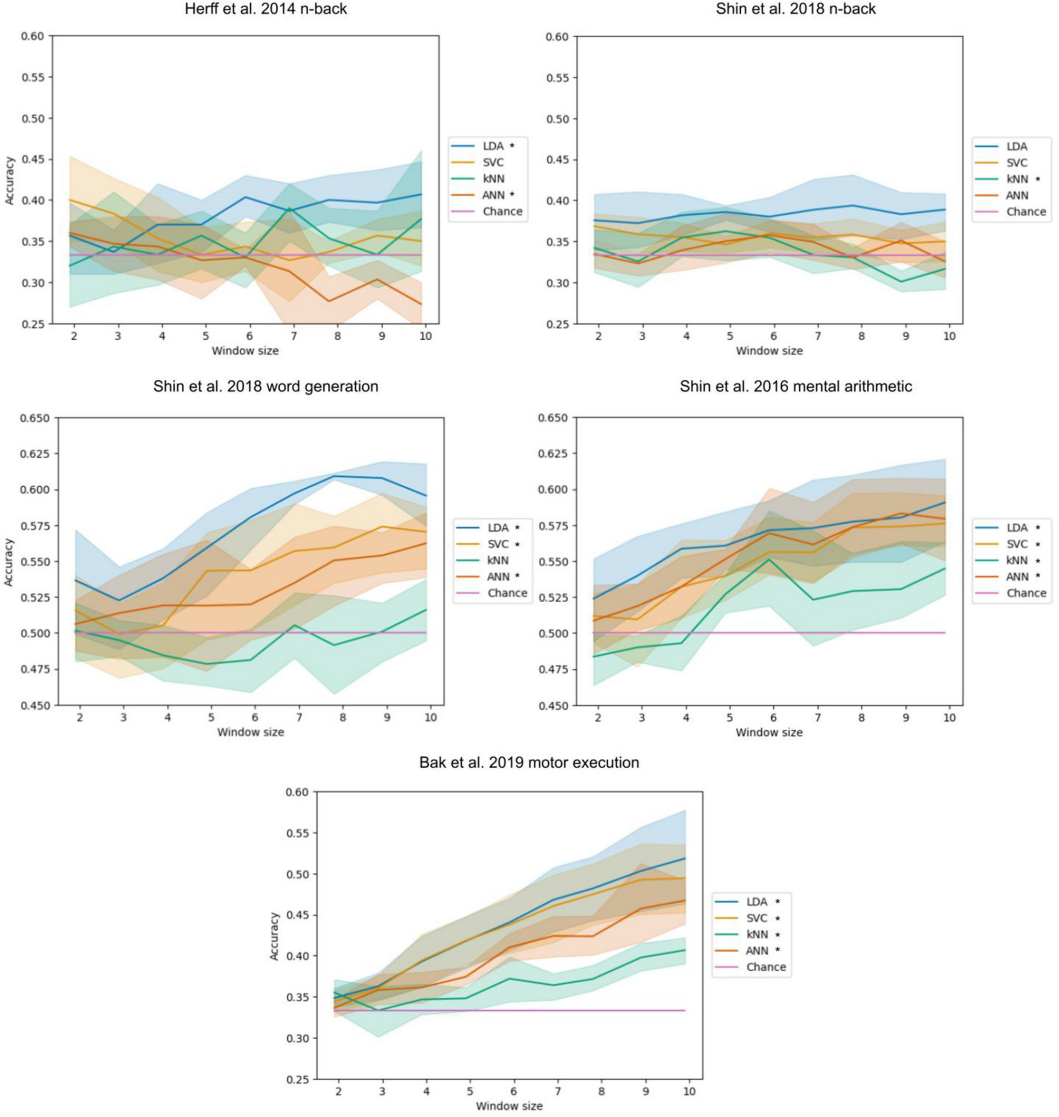
Accuracy with respect to the time window length. The bands represent the 95% confidence interval related to the variability on the five-fold outer cross-validation. Legend entries marked with an asterisk indicate a significant correlation at a 5% threshold.

The influence of the time window length used as input of the models on the classification accuracy of the LDA, SVC, kNN, and ANN can be seen (Figure 2). This took 38 h and 41 min to run.

For the Herff et al. (2014) dataset of n-back tasks, a significant positive correlation was found for the LDA (*p*-value of 0.004) with a correlation coefficient of 0.417 and a negative correlation for the ANN (*p*-value< 0.001) with a correlation coefficient of −0.513.

For the Shin et al. (2018) dataset of n-back tasks, a significant negative correlation was found for the kNN with a correlation coefficient of −0.319 (*p*-value of 0.033).

For the Shin et al. (2018) dataset of word generation tasks, significant positive correlations were found for the LDA, SVC, and ANN with *p*-values inferior or equal to 0.001. The correlation coefficients are 0.732, 0.585, and 0.462, respectively. Spearman tests were used for the LDA and the SVC because of the non-normality of distributions.

For the Shin et al. (2018) dataset of mental arithmetic tasks, significant positive correlations were found for the LDA, SVC, kNN, and ANN with p-values inferior to 0.001. The correlation coefficients are 0.510, 0.665, 0.504, and 0.646, respectively.

For the Bak et al. (2019) dataset of motor execution tasks, significant positive correlations were found for the LDA, SVC, kNN, and ANN with all p-values inferior to 0.001. The correlation coefficients are 0.803, 0.788, 0.618, and 0.836, respectively. A Spearman test was used for the LDA because of the non-normality of the distribution.

### 3.4. Generalised approach with sliding window

**Figure 3:**
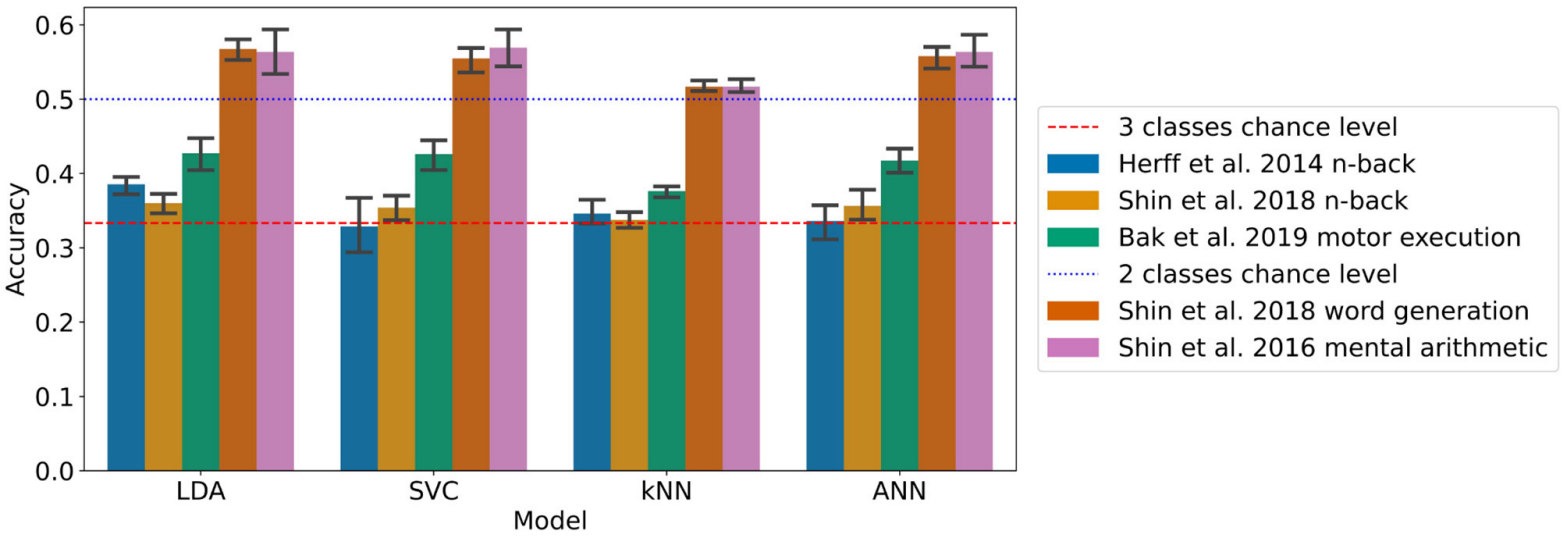
Accuracies of the models on each dataset with the generalised approach using a 2 s sliding time window (95% confidence intervals related to the variability on the five-fold outer cross-validation are displayed).

The results with the generalised approach using a 2 s sliding time window for the LDA, SVC, kNN, and ANN can be found in Figure 3 and Table 4. This took 22 h and 19 min to run.

**Table 4:** Accuracies of the models on each dataset with the generalised approach using a 2 s sliding time window.

**Dataset**	**Chance level**	**LDA**	**SVC**	**kNN**	**ANN**
Herff et al. (2014)	0.333	0.385^*^	0.329	0.346	0.336
n-back		(0.014)	(0.040)	(0.020)	(0.028)
Shin et al. (2018)	0.333	0.360^*^	0.354	0.337	0.356
n-back		(0.014)	(0.021)	(0.013)	(0.023)
Shin et al. (2018)	0.500	0.568^*^	0.555^*^	0.517^*^	0.558^*^
word generation		(0.016)	(0.019)	(0.008)	(0.017)
Shin et al. (2016b)	0.500	0.564^*^	0.569^*^	0.517^*^	0.564^*^
Mental arithmetic		(0.036)	(0.028)	(0.011)	(0.024)
Bak et al. (2019)	0.333	0.427^*^	0.426^*^	0.376^*^	0.417^*^
Motor execution		(0.024)	(0.023)	(0.009)	(0.019)

Fields marked with an asterisk indicate an accuracy significantly greater than chance level at a 5% threshold, the standard deviation on the five-fold outer cross-validation is in parenthesis.

For the n-back task datasets from Herff et al. (2014) and Shin et al. (2018), the accuracy was found significantly greater chance level (33.3%) for the LDA with *p*-values of 0.001 and 0.010, respectively, corresponding to accuracies of 38.5 and 36.0% respectively with 3 classes.

For the Shin et al. (2018) dataset of word generation tasks, significant differences compared to chance level (50%) were found for the LDA, SVC, kNN, and the ANN with *p*-values up to 0.008. The accuracies range from 51.7 to 56.8% with two classes.

For the Shin et al. (2016b) dataset of mental arithmetic tasks, significant differences compared to chance level (50%) were found for the LDA, SVC, kNN, and ANN with *p*-values ranging from 0.003 to 0.02 and accuracies ranging from 51.7 to 56.9% with two classes.

For the Bak et al. (2019) dataset of motor execution tasks, significant differences compared to chance level (33.3%) were found also for the LDA, SVC, kNN, and ANN with p-values inferior to 0.001. The accuracies range from 37.6% with the kNN to 42.7% with the LLDA.

A significant influence of the model on the classification accuracy was found for all the datasets except the Shin et al. (2018) dataset of n-back tasks. More specifically with pairwise t-tests, on the Herff et al. dataset of n-back tasks, the accuracy of the LDA was found significantly greater than the accuracy of the kNN. On the Shin et al. (2018) dataset of word generation tasks, the LDA accuracy was found significantly greater than all the other models with sliding window, and the kNN accuracy was found significantly lower than the SVC and ANN accuracies. Finally on the Bak et al. (2019) dataset of motor execution tasks, the kNN accuracy was found significantly lower than all the other models with sliding window.

A detail of the hyperparameters selected with grid search for each iteration of the outer cross-validation can be found in the Supplementary material as well as the results of the statistical tests.

### 3.5. Personalised approach

The results with the personalised approach which took 24 h and 58 min to run can be found in Table 5.

**Table 5:** Accuracies of the models on each dataset with the personalised approach.

**Dataset**	**Chance**	**LDA**	**SVC**	**kNN**	**ANN**	**CNN**	**LSTM**
Herff et al. (2014)	0.333	0.353^[1/10]^	0.350^[1/10]^	0.350^[4/10]^	0.373^[2/10]^	0.353^[0/10]^	0.360^[2/10]^
n-back		(0.099)	(0.065)	(0.128)	(0.076)	(0.034)	(0.099)
Shin et al. (2018)	0.333	0.360^[3/26]^	0.319^[3/26]^	0.329^[0/26]^	0.343^[2/26]^	0.317^[0/26]^	0.356^[1/26]^
n-back		(0.103)	(0.099)	(0.095)	(0.065)	(0.049)	(0.083)
Shin et al. (2018)	0.500	0.588^[7/26]^	0.562^[6/26]^	0.546^[4/26]^	0.562^[8/26]^	0.554^[8/26]^	0.549^[6/26]^
word generation		(0.090)	(0.099)	(0.068)	(0.088)	(0.089)	(0.076)
Shin et al. (2016b)	0.500	0.633^[16/29]^	0.594^[12/29]^	0.563^[10/29]^	0.585^[10/29]^	0.601^[8/29]^	0.608^[10/29]^
mental arithmetic		(0.107)	(0.104)	(0.080)	(0.087)	(0.099)	(0.092)
Bak et al. (2019)	0.333	0.513^[18/30]^	0.444^[14/30]^	0.380^[7/30]^	0.387^[6/30]^	0.402^[8/30]^	0.446^[15/30]^
motor execution		(0.140)	(0.100)	(0.084)	(0.078)	(0.073)	(0.107)

The number of participants out of the total number of participants of each dataset having an accuracy significantly greater than chance level at a 5% threshold is presented in brackets for each model. The standard deviation on participants is in parenthesis.

The detailed statistical analysis for each subject can be found in the Supplementary material. The general trend however follow the one of the generalised approach, with more results significantly different from chance level with the dataset from Bak et al. (2019) of motor execution tasks and the dataset from Shin et al. (2016b) of mental arithmetic. Moreover, the results are very subject dependant which is also shown by the high values of standard deviation as seen in Table 5.

## 4. Discussion

### 4.1. Generalised approach (*RQ2*)

Regarding the benchmarkings of popular machine learning models on the five datasets, the first thing that the results show is that the performances are rather low overall (and typically lower than reported in some published works), which can be explained in multiple ways. First of all, the methodology prevents any kind of optimisation of the hyperparameters on the test set, which make the results representative of what would happen with actual unseen data in the case of a real-life BCI application. Secondly, the models evaluated in this work present largely optimised baseline models that can be used in comparison for future machine learning developments and new datasets. More complex deep learning architectures, for example, could eventually help improve the performance. Also, the signal processing and the extraction of features have not been the focus of this work, and using approaches more personalised to each case would likely lead to better results for that case. Furthermore, most of the datasets found that meet our criteria are comparatively small and research with more examples could be beneficial as we discuss in the following subsection, especially for the deep learning models. We hope that researchers will contribute both more advanced models and larger datasets to this benchmarking framework, as part of a shared community drive toward making clear advances.

Another finding is that the performances appear different with the type of task dataset. Indeed the performances on the Bak et al. (2019) dataset of motor execution tasks are higher than on the other datasets with three classes (Herff et al., 2014; Shin et al., 2018 datasets of n-back tasks). An explanation could lie in the nature of the tasks: the brain activity elicited by motor execution is easier to highlight than the brain activity elicited by mental workload tasks which rely on higher level brain processes (Friedman and Robbins, 2021). We also see that the datasets with two classes of word generation (Shin et al., 2018) and mental arithmetic (Shin et al., 2016b) also have classification accuracies generally greater than chance level, which may be explained by the fact that both are task detection datasets (baseline vs. task) as opposed to classifying the level of a task as done with the n-back datasets.

It also appears that the variability with different test sets can sometimes be quite high for some models and some datasets, which could mean that it is more difficult to preform classification on some unseen subjects compared to others, probably in cases where their data looks different than the one from participants in the training set.

Regardless, one of the outcomes is that in most cases the kNN seems to underperform compared to other models, while the other standard machine learning models with feature extraction, especially the LDA, do not perform worst than deep learning methods using raw data (CNN and LSTM). Standard machine learning models (LDA and SVC) then remain a relevant choice especially because of their low computational complexity. This goes in the same direction as other works such as Hennrich et al. (2015) showing that deep learning methods achieve comparable accuracies to conventional methods.

### 4.2. Influence of training set size on generalised approach (*RQ3a*)

Only two significant correlations of the accuracy to the percentage of training examples have been found out of the 30 tested in total, which is quite low. Those are a negative correlation with a kNN model and a positive correlation with a CNN model. This positive correlation goes with the tendency of deep learning models to perform better with bigger datasets, however in our case it has only been highlighted with one deep learning model on the motor execution dataset from Bak et al. (2019). Even though this dataset is amongst those with the most examples with 750 per class in total (leading in the training set to 600 examples per class with 100% and 300 per class with the minimum studied here of 50% of training data), this trend is not shown with the biggest dataset containing 870 examples per class (mental arithmetic from Shin et al., 2016b). It is likely that even with 100% of the training data, the training sets remains very small for all the datasets, especially for classification with deep learning. Indeed with three classes the number of trainable parameters is 155, 491, and 17635 for the ANN, CNN, and LSTM, respectively (150, 480, and 17,618, respectively with two classes) which is to relate to the total number of examples in the datasets between 300 and 2,250. The influence of the dataset size would be interesting to study further with bigger fNIRS datasets to see if there is really a clear effect with deep learning models.

### 4.3. Influence of window length on generalised approach (*RQ3b*)

Based on the results, it appears that overall the length of the time window used as input for classification does have an influence on the performance of models using feature extraction, as the correlations show.

Each of our correlations are positive except 2 negative correlations which were found on the n-back datasets. Those negative correlations are however hard to interpret with certainty since the accuracy on those n-back tasks remains very low overall and in most cases is not significantly higher than chance level as seen with the generalised approach with 10 s epochs. Also, precautions should be taken for short time windows on n-back tasks because they do not enable to express the whole difficulty of the task since a new stimulus is presented every 2 s from the beginning of the task and n stimuli are required to reach the actual task demand with n-back.

All other correlations were positive, for most of the models with which the classification accuracy was significantly greater than chance level, which makes us believe that bigger time window actually benefits classification accuracy. This is especially striking for the Bak et al. (2019) dataset of motor execution tasks as seen in Figure 2. This benefit of longer time windows is likely explained by the duration of the hemodynamic response, being around 4–6 s (Buxton et al., 2004), making shorter time window too small to capture relevant changes.

### 4.4. Generalised approach with sliding window (*RQ3c*)

Using a sliding approach has two main advantages. First, it enables us to multiply the number of examples that can be used as input for the classifiers. Secondly, it enables us to make a prediction on the class every 2 s which can be useful in the context of a BCI.

With the models using feature extraction, the accuracies using a 2 s sliding window are found significantly greater than chance level in the same cases as the generalised approach with non-sliding 10 s epochs. Here again, the kNN seems to underperform in most cases compared to other models. For all the models tested here with sliding window, results appear overall slightly lower than those with the 10 s epochs, even though the performances are higher than those obtained with a non-sliding window of 2 s which are around chance level. It may be possible that the decrease in performance observed with smaller time windows as described previously is compensated by the increase in training examples. Also, even though previous work has shown potential at classifying fNIRS data using short time windows from the task trigger onset, hence focusing on the initial dip part of the hemodynamic response (Zafar and Hong, 2017; Khan and Hong, 2021), it may be possible that later segments are more useful to discriminate between conditions. Further studies would need to be conducted to compare different centering of shorter time windows as an extension of *RQ3b* and *RQ3c*. Regardless, such sliding window approaches remain relevant for BCI applications when it is desired to have predictions made regularly in real-time.

### 4.5. Personalised approach (*RQ3d*)

The results highlight a very high variability across subjects, and the average results are not much different from the generalised approach. This personalised approach, though, seems to produce relatively high results on the Shin et al. (2016b) dataset of mental arithmetic tasks. It also appears that the LDA model with feature extraction preforms quite well on most of the datasets.

These results with our methodology however remain low compared to what can be found in existing literature. Indeed, most of the papers which have proposed a classification using machine learning on the same datasets we used are using a personalised approach. For example, Herff et al. (2014) reached slightly higher accuracies on their n-back dataset with a LDA using the slope on each channel and on a slightly longer time window (15 s) than our experiments, with 44.0% on average with 10-fold cross-validation (meaning more training data than five-fold) compared to 35.3% with our baselines. Shin et al. (2016b) with shrinkage LDA using average and slope for each channel on 3 s moving windows reached 80.7% with HbR and 83.6% with HbO with five-fold cross-validation on their mental arithmetic dataset, compared to 63.3% with our baselines. Bak et al. (2019) with linear SVC using the average for each channel on 5 s moving time windows reached an average accuracy of 70.4% on their motor execution dataset, compared to 44.4% in our results. Finally, compared to our results around chance level with a CNN using temporal convolutions, Saadati et al. (2019) reached an accuracy of 82% on average on the Shin et al. (2018) dataset of n-back tasks with a CNN using spatial convolutions, however they did not describe how the dataset was split into training, validation and test sets. All those existing results show the difficulty of comparison when lacking standardised methodology, also when some methods cannot be applied to other datasets due to constraints from the experimental design (e.g., length of time windows, number of channels). We hope that BenchNIRS will allow future work with notably high accuracy to now more easily demonstrate their advances for improved performance in a comparable way, and more easily check against common mistakes.

Another point of discussion is that the results of the personalised approach do not, unfortunately, give the opportunity to draw higher level conclusions, as every subject only took part for one session in all the datasets, making it impossible to determine whether the results are related to session specific factors or subject specific factors.

Finally, it should be noted that such personalised models are hardly usable in the context of a real-life BCI, especially if they require model training on each session. Indeed, in the case where the results are obtained with a five-fold outer cross-validation, it means that the model requires 80% of the data for training, meaning that the majority of the time would be dedicated to calibrating the BCI with the subject rather than using it. This is also why our work mainly focuses on generalised approaches which can be applied more easily in real-life BCI settings. Also, this relates to why the data is not normalised with a min-max feature scaling or standard score: computing those on the whole dataset would bias the results on the test set (because test data would have been seen already to normalise) and is not possible in real time, and computing those with the mean or min-max computed on the training set only could shift the distribution of the test set if it is very different from the training set (if the mean or min-max are quite different on the test set than the training set).

### 4.6. Limitations and future work

Our work provides novel insights into factors influencing the performance of machine learning classifiers using fNIRS data, in the hope to help readers looking for the model that would best suit their needs. This is however an entry point into the benchmarking of machine learning for fNIRS. Therefore, some limitations remain for future work to address.

First of all there are limitations due to the datasets used in this framework. Most of the datasets contain a limited amount of data which is critical for the performance of some machine learning models, especially those having a lot of parameters. Datasets with more subjects could be added to the framework in the future, such as Huang et al. (2021), however the lower sampling frequency of this dataset in particular would further affect the comparison across the other datasets taken at a higher sampling frequency. Another point is that none of the datasets have used an fNIRS device with short-separation channels, which limits the extent of noise removal that can be performed. Indeed, having such short-separation channels for each dataset would have helped removing artifacts due to superficial hemodynamics reflecting systemic physiological changes (Brigadoi and Cooper, 2015; Sato et al., 2016). Also, the datasets were based on recordings from participants that took part in only one session, which limits the conclusions that can be drawn when it comes to studying participant or session specificity. Those constraints come with the limited availability of open access fNIRS datasets, and future work would consist of extending the framework to other newly published open access datasets addressing those issues. Indeed, we welcome dataset contributions (see Section 4.8).

Secondly, the performance of our models may be limited by the compromises that had to be made for the sake of comparison between datasets with different experimental design and different equipment. For example, we had to accommodate for the sampling frequency (which is why downsampling has been performed), the number of channels (which is why region of interest averaging has been performed) and epoch duration (which is why epoch cropping was performed).

Also, the work is also potentially limited by the signal processing and feature extraction used. The signal processing selected in our framework follows a recommended approach with TDDR and bandpass filtering to remove signal noise. This choice was made because the comparison of signal processing is not the focus here and has been studied in other published works such as Brigadoi et al. (2014) and Pinti et al. (2018b). However, the benchmarking could be extended by involving different and more advanced signal processing techniques. Indeed, approaches more tailored to each task would be more efficient at removing signal noise for that dataset, but a classic approach was used here for comparison. Similar remarks can be made regarding the feature extraction. Further, as the datasets involved devices with different numbers of channels, we needed to average the channels in regions of interest for the sake of comparability of models, but more work could be done regarding the spatiality of the brain activity.

Finally, each of the machine learning models used in our study could have been developed and tweaked in different ways. We decided to implement common baseline models to act as a starting point for benchmarking, however, more complex models could be implemented. For example here, shrinkage LDA could be compared to standard LDA. Also, the architectures of the deep learning models have been chosen keeping into consideration the input data dimensions and the number of training examples, but they could be more extensively optimised, and finding optimal architectures could be the matter of future works. This could include, for example, architectures valuing more the spatiality of signals. Similarly, different kernels could be tested for the SVC for example. Our work, however, means that is now possible to implement such more-advanced models in future work, using our framework, and for them to be validated robustly with the recommended checklist of methodological steps.

### 4.7. Recommendations toward best practices for machine learning with fNIRS

First and foremost, we would like to encourage fNIRS machine learning researchers to follow fNIRS specific guidelines already described in important previous work for signal processing with Pinti et al. (2018b) and Santosa et al. (2020), but also best practices for publications with Yücel et al. (2021). Further to these, to answer *RQ1* and in line with other fields of application (Mongan et al., 2020), we provide recommendations that we believe important when using machine learning for classification from fNIRS data, based upon the practice of making our benchmarked comparisons above. Some of these recommendations have become standard process in machine learning, but aspects are often missed in recent machine learning papers within the fNIRS community.

The first recommendations are methodology related:

- plan classes before designing the experiment (to avoid using return to baseline as control baseline task);
- use nested cross-validation, also called double cross-validation with the outer cross-validation (leaving out the test sets) for evaluation and the inner cross-validation (leaving out the validation sets) for the optimisation of models;
- optimise the hyperparameters (with grid-search for instance) on validation sets;
- use the test sets for evaluation and nothing else (no optimisation should be performed with the test set);
- create the training, validation and test sets in accordance with what the model is hypothesised to generalise (e.g., unseen subject, unseen session, etc.), thanks to group k-fold cross-validation for example;
- pay attention to not include test data when performing normalisation;
- take extra care to not have any of the sets overlap (training, validation, and test sets), the test set used to report results more than anything must consist of unseen data only;
- pay attention to class imbalance (using metrics more appropriate than accuracy such as F1 score for example);
- perform a statistical analysis to find significance of the results when comparing results to chance level and classifiers to each other.

Where relevant, these points have been implemented in our framework and we therefore encourage researchers to use it for time saving and reproducibility purposes.

The second recommendations are related to the reporting:

- describe what data is used as input of the classifier and its shape;
- describe the number of input examples in the dataset;
- describe the details of the cross-validations implementations;
- describe the details of each model used including the architecture of the model and every hyperparameter;
- describe which hyperparameters have been optimised and how;
- clearly state the number of classes and the chance level;
- provide all necessary information related to the statistical analysis of the results, including the name of the tests, the verification of their assumptions and the *p*-values.

Finally, we invite researchers to have a look at guidances from the machine learning community regarding reproducibility^5^ and research code publication.^6^

We hope our recommendation list can act as a starting point for the community to contribute to a more exhaustive checklist for the field of machine learning applied to fNIRS BCIs (see text footnote 2).

### 4.8. Description of the framework

To give concrete material to answer *RQ1*, the framework developed and used for this work called BenchNIRS is made available as an online git repository.^7^ BenchNIRS works on Python 3 and the application programming interface (API) enables researchers to customise the analysis pipeline, ranging from data loading to machine learning classification.

#### 4.8.1. Datasets

Functions to load 5 open access fNIRS datasets are available to the user: n-back tasks from Herff et al. (2014), n-back tasks from Shin et al. (2018), word generation tasks from Shin et al. (2018), mental arithmetic tasks from Shin et al. (2016b), and motor execution tasks from Bak et al. (2019). This enables us to load epochs as designed in the original studies.

#### 4.8.2. Signal processing

The framework implements various signal processing techniques that can be adjusted by the user: motion artifact correction, baseline correction, use of original channels or region of interest channel averaging, filtering (low-pass, high-pass, and band-pass) with adjustable edge frequencies and order. The epochs can be cropped and the option for a sliding time window can be selected.

#### 4.8.3. Feature extraction

Features can be extracted including the mean, the standard deviation, and the slope of the linear regression.

#### 4.8.4. Machine learning methodology

Nested cross-validation is implemented: evaluation is performed on the outer cross-validation and the inner cross-validation is used for hyperparameter optimisation. One can use this methodology with a generalised or personalised approach. The training set size can be reduced to study the influence of its size. Metrics including the accuracy, the precision, the recall, and the F1 score can be produced. Graphs are drawn (with a color blind palette for accessibility) including training graphs (accuracy and loss), confusion matrices, as well as box plots and graphs with 95% confidence intervals for overall results. Default models can be trained including the LDA, SVC, kNN, ANN, CNN, and LSTM presented in this manuscript, but customised models can also be used simply with this methodology.

#### 4.8.5. Statistical analysis

Examples of statistical analysis produced under the form of tables are provided including comparison to chance level, comparison of models, correlation to training size, and time window length.

#### 4.8.6. Community contributions

The repository also contains a checklist of recommendations for machine learning with fNIRS (see text footnote 2). The repository will be open to community contribution in order to add support for new open access datasets, improve the checklist, the implementation of the machine learning methodology, or the production of results and figures. Guidance on how to contribute can be found on the repository page.

Furthermore, we encourage researchers to use the framework if they wish to compare the results obtained with their machine learning models on the datasets supported by BenchNIRS with the proposed methodology.

## 5. Conclusion

Our work has introduced a framework called BenchNIRS for benchmarking of machine learning with fNIRS enabling researchers to robustly validate classification results on five open access datasets published by the community. This framework is used to perform the analysis of six baseline machine learning models: LDA, SVC, kNN, ANN, CNN, and LSTM. We also used BenchNIRS to produce results with different approaches: generalised, generalised with a sliding window, and personalised. Further we studied the influence of the training set size as well as the time window length (from 2 to 10 s) on the model performances.

Where most published research has studied specific models applied to specific datasets, we show with our initial benchmarking that no baseline model (standard machine learning or deep learning) statistically stands out consistently compared to others when applied across datasets except the LDA, hence remaining a strong choice despite its simplicity. Furthermore, we found no consistent influence of the training size on the classification accuracy. Finally, our results show that when models using common feature extraction techniques (mean, standard deviation, slope) perform greater than chance level, they benefit from longer time windows.

We invite the fNIRS community to use our framework when performing classification with their own machine learning models for a convenient evaluation on open access data and a comparison to our initial baseline results. We welcome contributions to extend and strengthen the guidelines that we propose as well as the implementation of the machine learning methodology.

## Data availability statement

Publicly available datasets were analysed in this study. This data can be found at: http://www.csl.uni-bremen.de/CorpusData/download.php?crps=fNIRS; http://doc.ml.tu-berlin.de/simultaneous_EEG_NIRS/NIRS/NIRS_01-26_MATLAB.zip; http://doc.ml.tu-berlin.de/hBCI; https://figshare.com/ndownloader/files/18069143.

## Ethics statement

Ethics approval was stated in the papers which produced the datasets used in our work. The participants provided their written informed consent to participate in this study.

## Author contributions

JB: methodology, software, and writing. JC: machine learning methodology review, software review, and manuscript review. AL: signal processing methodology review and manuscript review. MV: machine learning methodology review and manuscript review. MW: methodology and writing. All authors contributed to the article and approved the submitted version.

## Footnotes

^1^ https://openfnirs.org/data/

^2^ https://gitlab.com/HanBnrd/benchnirs/-/blob/main/CHECKLIST.md

^3^We choose not to call out specific papers and authors, but rather highlight things that researchers should look out for.

^4^ https://github.com/HanBnrd/NIRSimple

^5^ https://www.cs.mcgill.ca/~jpineau/ReproducibilityChecklist.pdf

^6^ https://github.com/paperswithcode/releasing-research-code

^7^ https://gitlab.com/HanBnrd/benchnirs
