# Supplementary material for: Benchmarking framework for machine learning classification from fNIRS data
Source: Front Neuroergon. 2023 Mar 3;4:994969. doi: 10.3389/fnrgo.2023.994969 (PMC10790918; doi:10.3389/fnrgo.2023.994969)
Supplement: Supplementary file 1 [file Data_Sheet_1.zip › supplementary/supplementary1_regions_interest.pdf]

MAPS OF THE REGIONS OF INTEREST

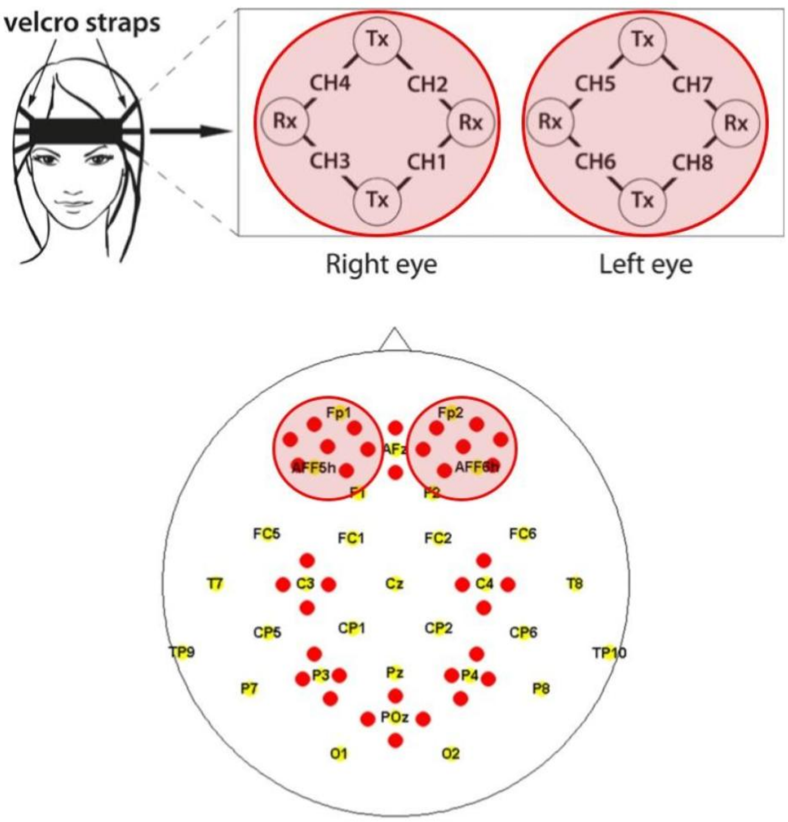

Figure 1. Maps of the regions of interest as circled in the red areas. *Top*: n-back dataset from Herff et al. 2014, fNIRS channels are represented by black lines with *CH* labels, each region of interest was an averaging of 4 channels. *Bottom*: n-back and word generation datasets from Shin et al. 2018, fNIRS channels are represented by red dots, each region of interest was an averaging of 7 channels.

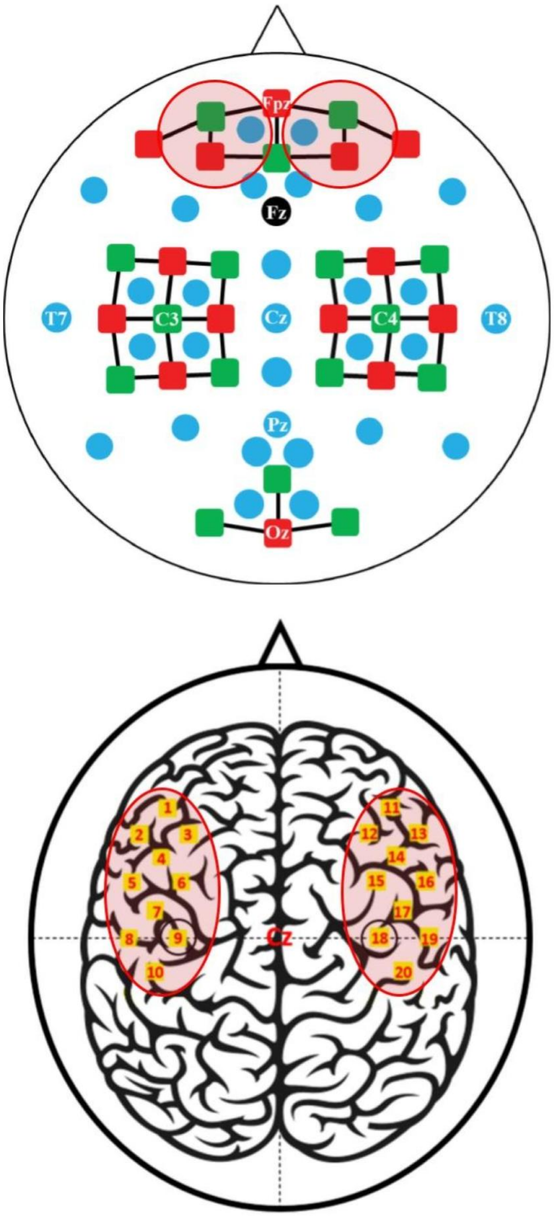

Figure 2. Maps of the regions of interest as circled in the red areas. *Top*: mental arithmetic dataset from Shin et al. 2016, fNIRS channels are represented by black lines between red (sources) and green (receptor) squares, each region of interest was an averaging of 4 channels. *Bottom*: motor execution dataset from Bak et al. 2019, fNIRS channels are represented by yellow squares, each region of interest was an averaging of 10 channels.
