# Supplementary material for: Benchmarking framework for machine learning classification from fNIRS data
Source: Front Neuroergon. 2023 Mar 3;4:994969. doi: 10.3389/fnrgo.2023.994969 (PMC10790918; doi:10.3389/fnrgo.2023.994969)
Supplement: Supplementary file 1 [file Data_Sheet_1.zip › supplementary/supplementary2_confusion_matrices.pdf]

CONFUSION MATRICES

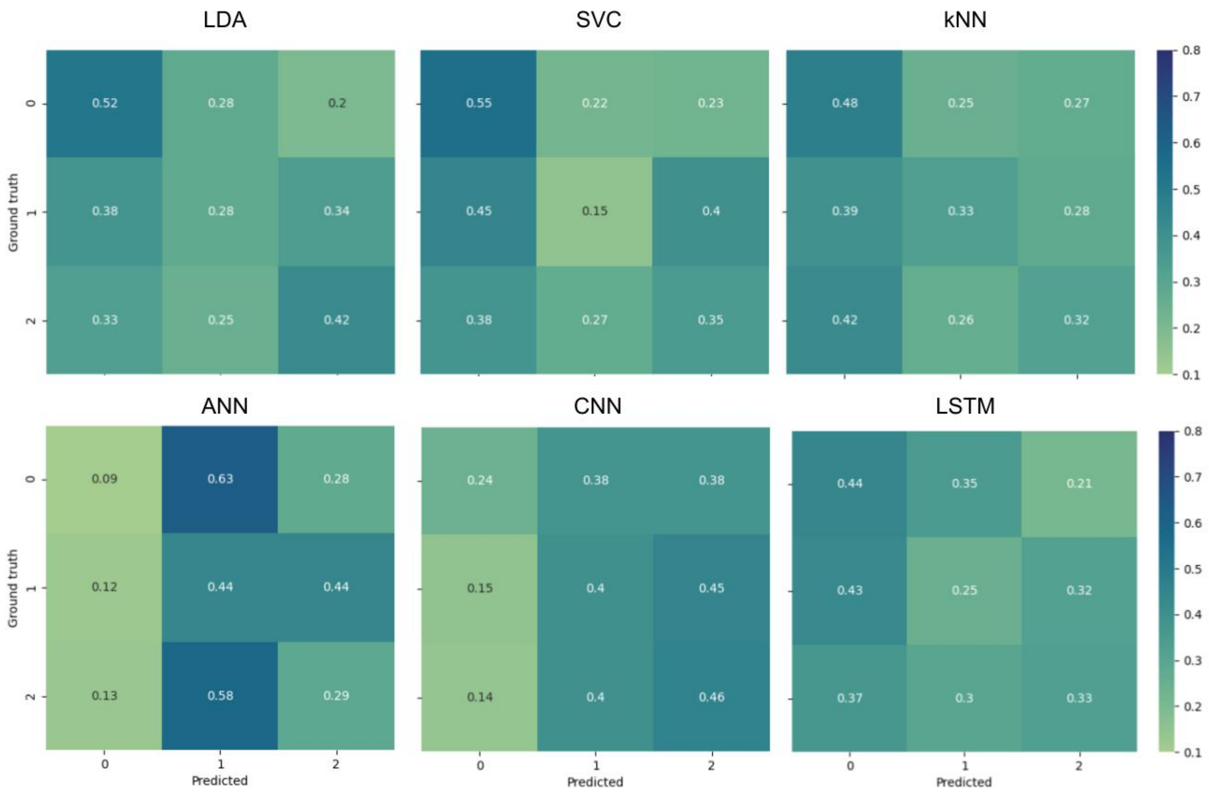

Figure 1. Confusion matrices for the generalised approach on the Herff et al. 2014 dataset of n-back tasks. Classes 0, 1 and 2 are 1-, 2- and 3-back respectively.

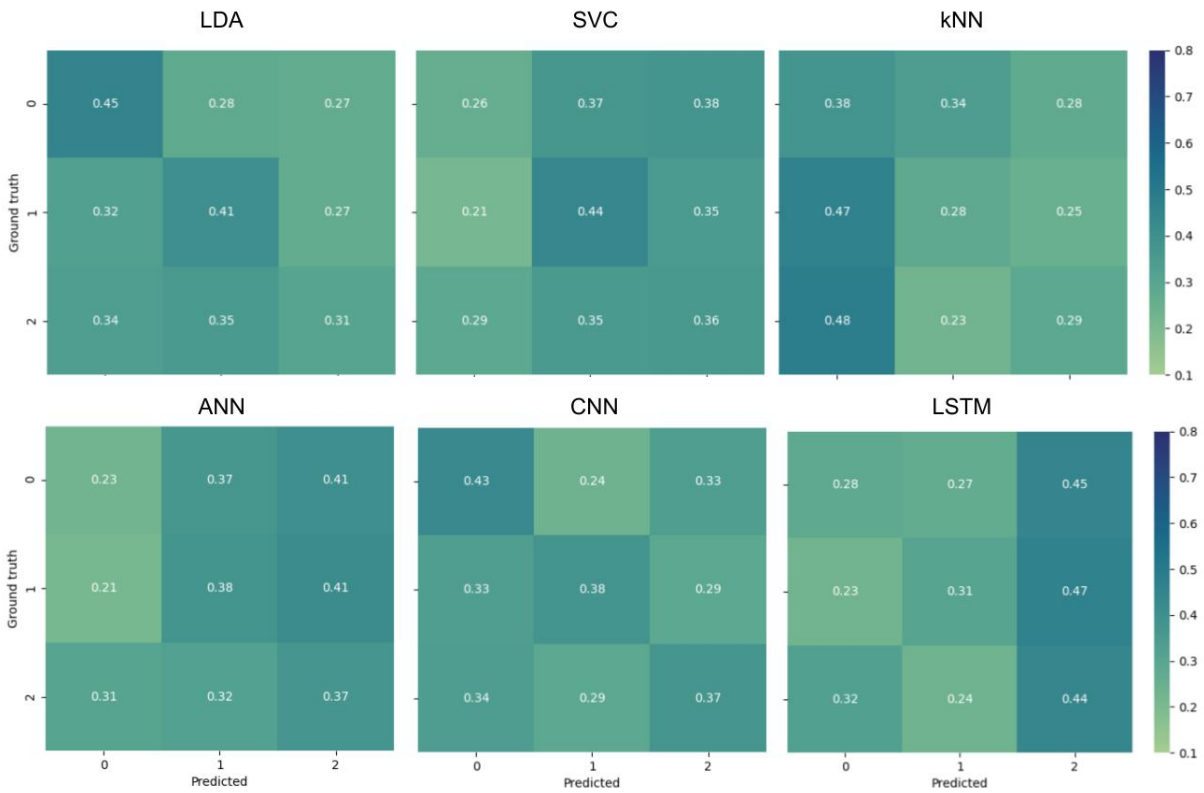

Figure 2. Confusion matrices for the generalised approach on the Shin et al. 2018 dataset of n-back tasks. Classes 0, 1 and 2 are 0-, 2- and 3-back respectively.

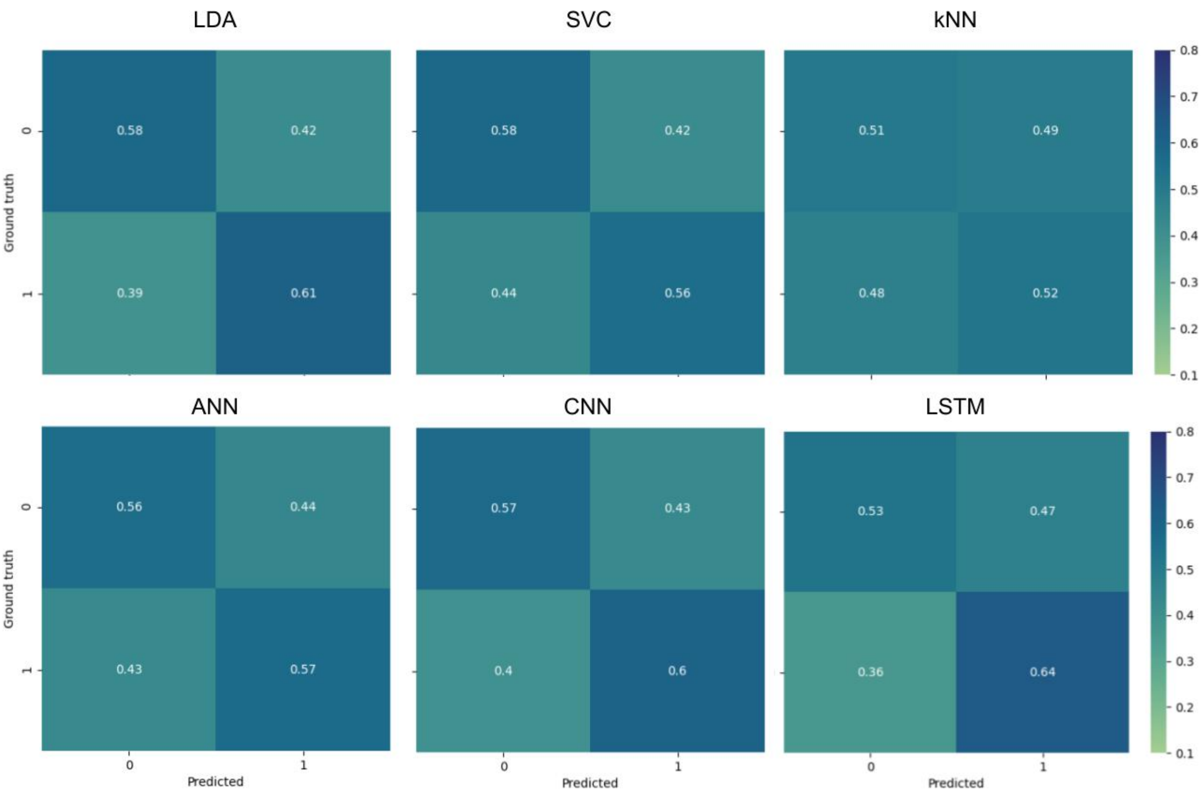

Figure 3. Confusion matrices for the generalised approach on the Shin et al. 2018 dataset of word generation tasks. Classes 0 and 1 are baseline task and word generation respectively.

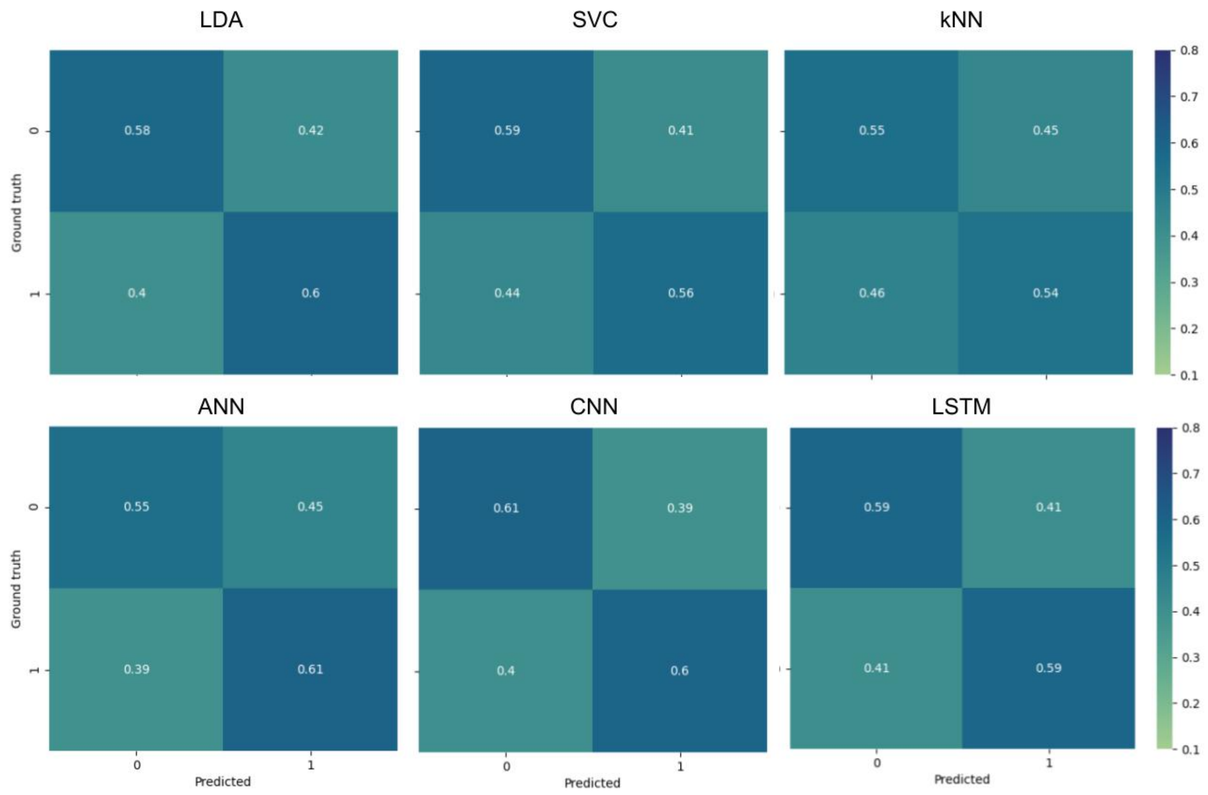

Figure 4. Confusion matrices for the generalised approach on the Shin et al. 2016 dataset of mental arithmetic tasks. Classes 0 and 1 are baseline task and mental arithmetic respectively.

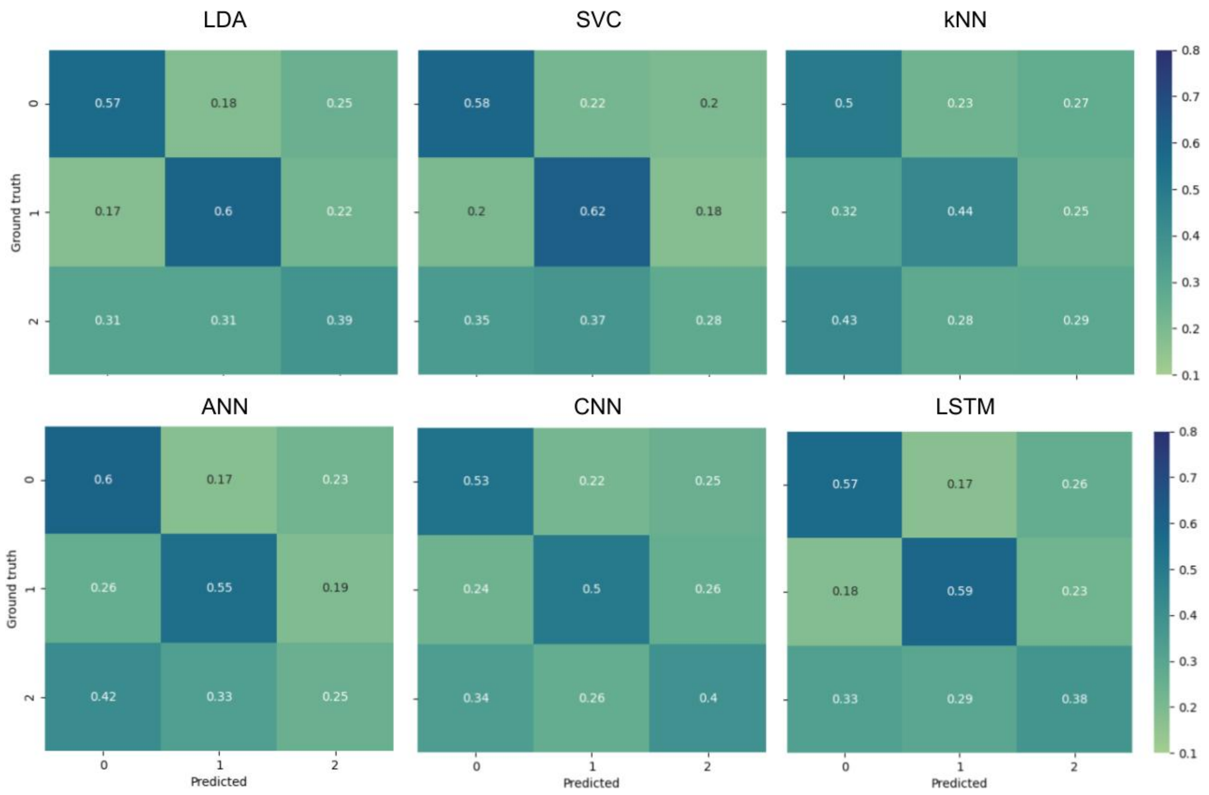

Figure 5. Confusion matrices for the generalised approach on the Bak et al. 2019 dataset of motor execution tasks. Classes 0, 1 and 2 are right hand finger tapping, left hand finger tapping and foot tapping respectively.
