# Supplementary figures and images for: Benchmarking framework for machine learning classification from fNIRS data
Source: Front Neuroergon. 2023 Mar 3;4:994969. doi: 10.3389/fnrgo.2023.994969 (PMC10790918; doi:10.3389/fnrgo.2023.994969)

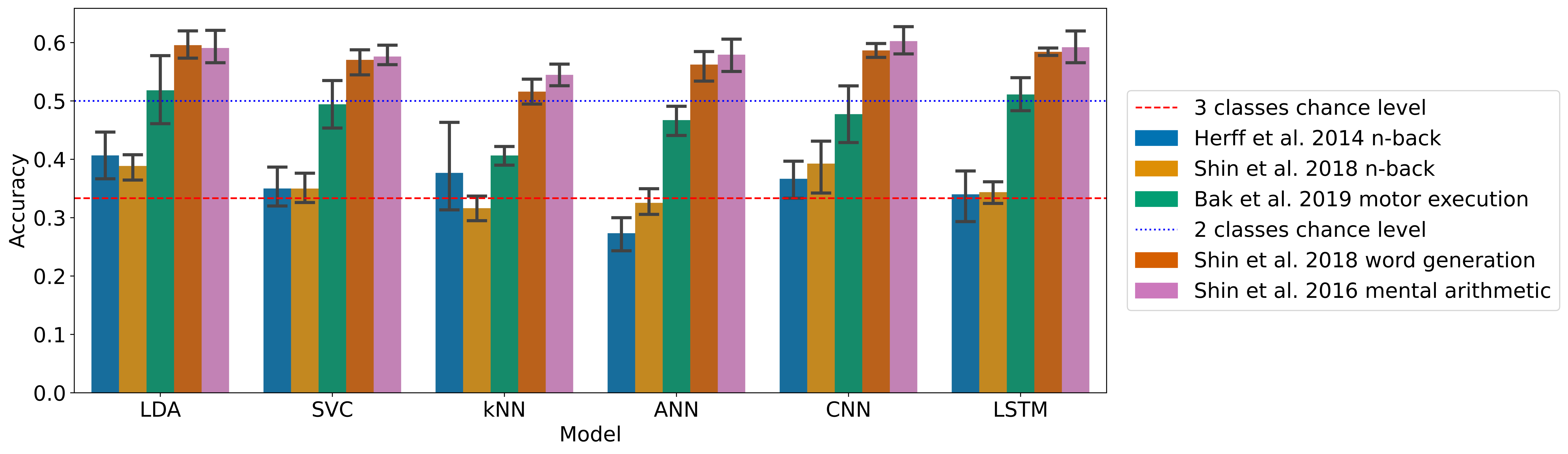

Supplement: Supplementary file 2 [file Data_Sheet_2.zip › benchnirs-v1.0/example.png]

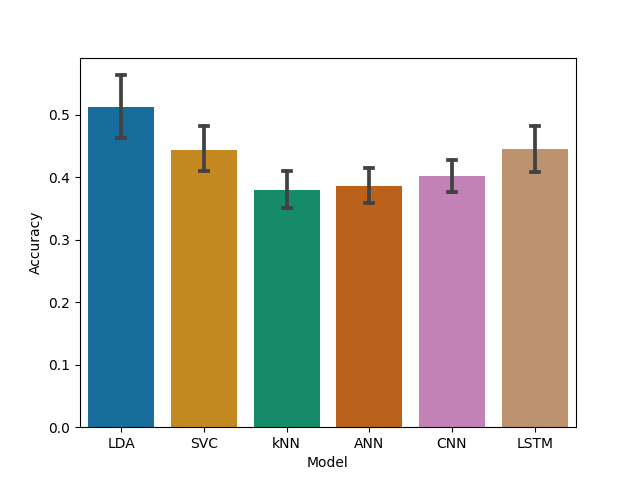

Supplement: Supplementary file 2 [file Data_Sheet_2.zip › benchnirs-v1.0/results/personalised/bak_2019_me_summary.png]

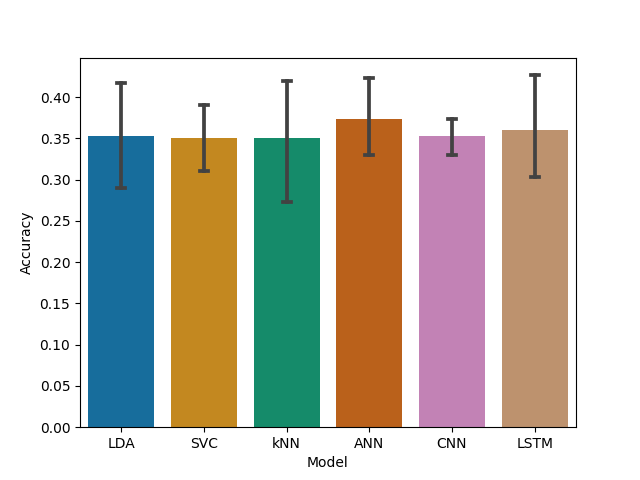

Supplement: Supplementary file 2 [file Data_Sheet_2.zip › benchnirs-v1.0/results/personalised/herff_2014_nb_summary.png]

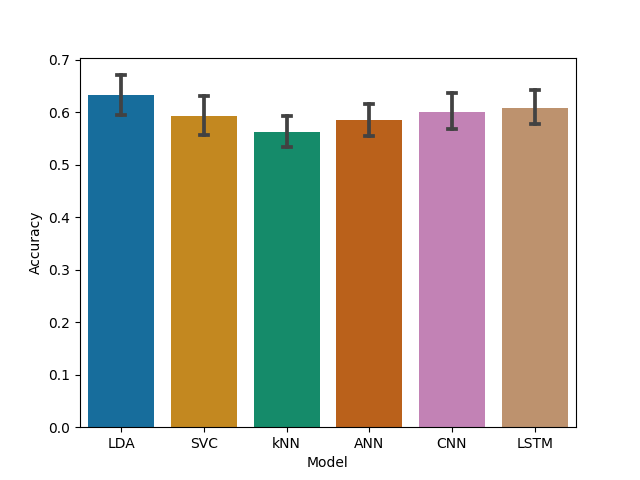

Supplement: Supplementary file 2 [file Data_Sheet_2.zip › benchnirs-v1.0/results/personalised/shin_2016_ma_summary.png]

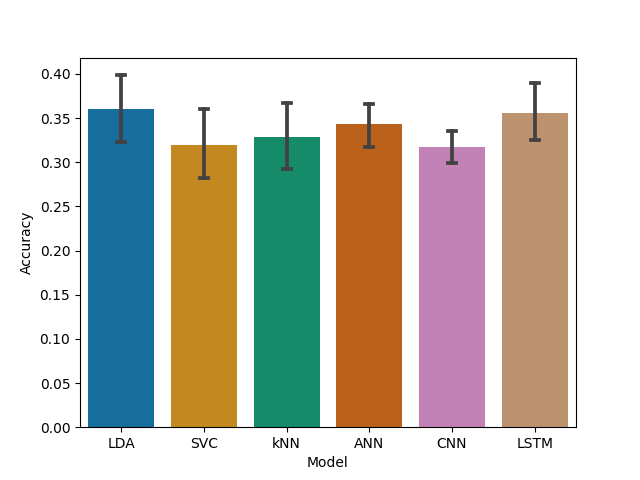

Supplement: Supplementary file 2 [file Data_Sheet_2.zip › benchnirs-v1.0/results/personalised/shin_2018_nb_summary.png]

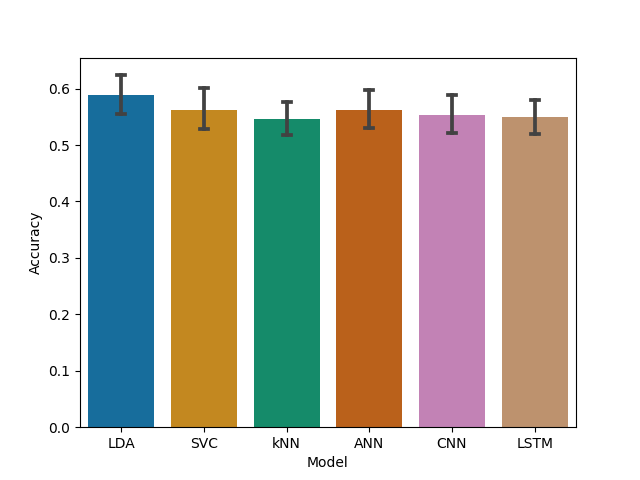

Supplement: Supplementary file 2 [file Data_Sheet_2.zip › benchnirs-v1.0/results/personalised/shin_2018_wg_summary.png]

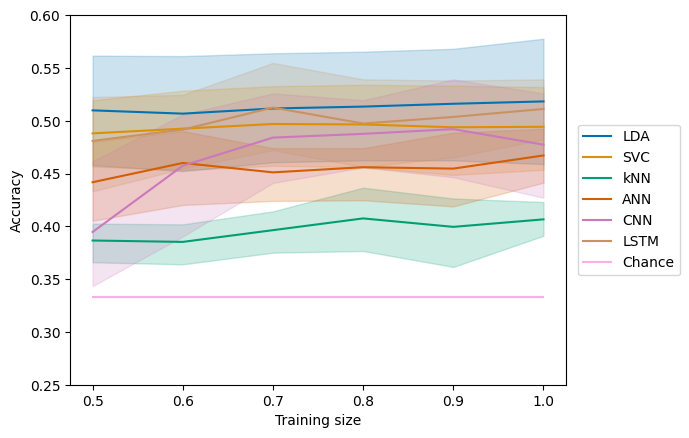

Supplement: Supplementary file 2 [file Data_Sheet_2.zip › benchnirs-v1.0/results/training_size/bak_2019_me_summary.png]

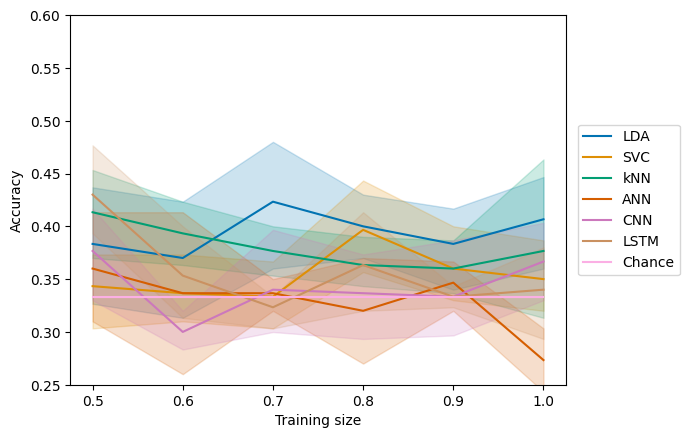

Supplement: Supplementary file 2 [file Data_Sheet_2.zip › benchnirs-v1.0/results/training_size/herff_2014_nb_summary.png]

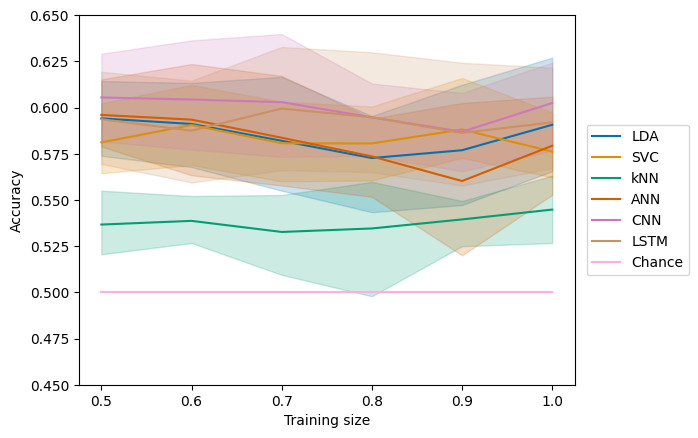

Supplement: Supplementary file 2 [file Data_Sheet_2.zip › benchnirs-v1.0/results/training_size/shin_2016_ma_summary.png]

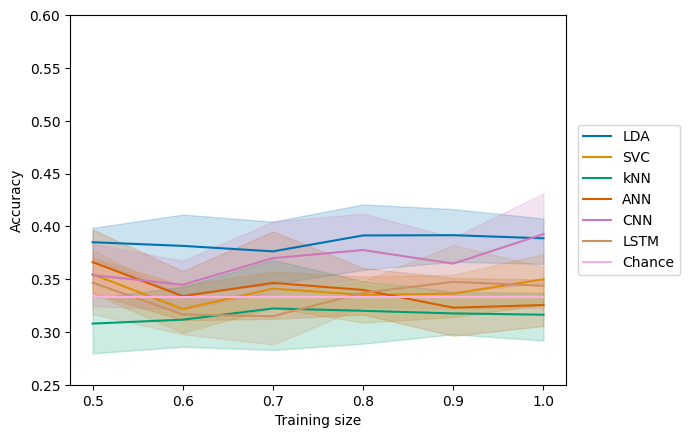

Supplement: Supplementary file 2 [file Data_Sheet_2.zip › benchnirs-v1.0/results/training_size/shin_2018_nb_summary.png]

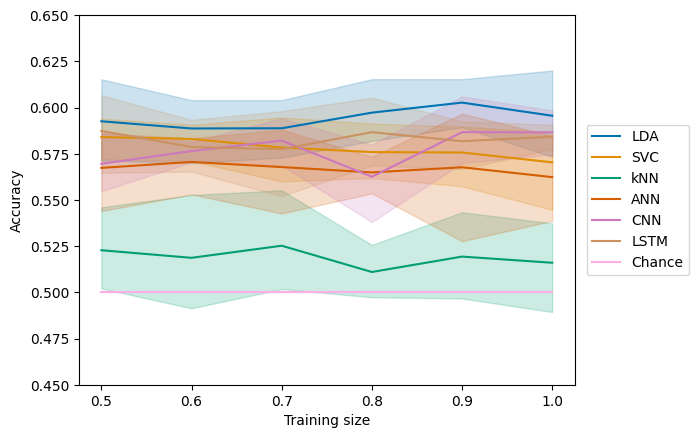

Supplement: Supplementary file 2 [file Data_Sheet_2.zip › benchnirs-v1.0/results/training_size/shin_2018_wg_summary.png]

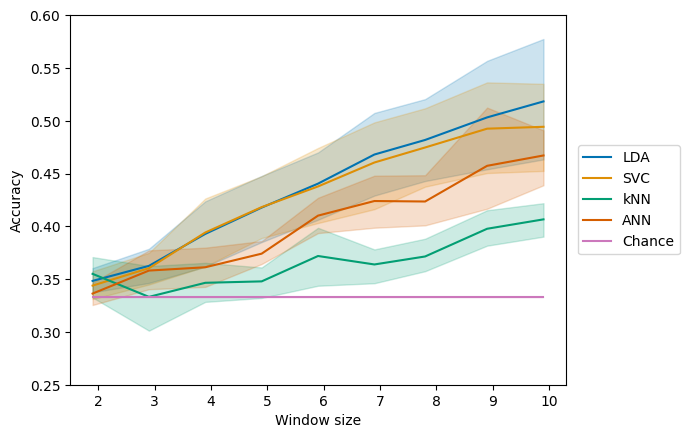

Supplement: Supplementary file 2 [file Data_Sheet_2.zip › benchnirs-v1.0/results/window_size/bak_2019_me_summary.png]

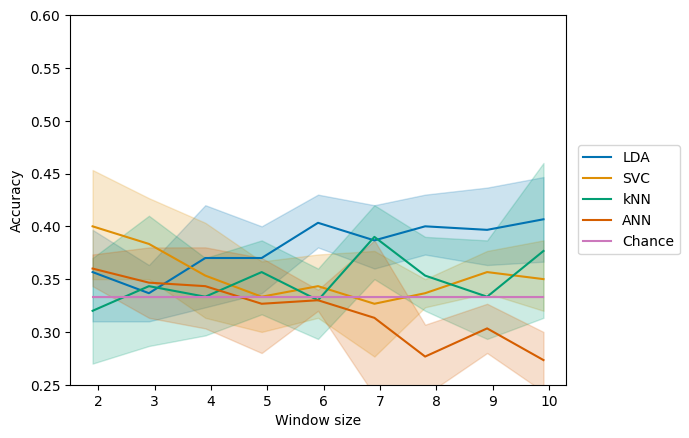

Supplement: Supplementary file 2 [file Data_Sheet_2.zip › benchnirs-v1.0/results/window_size/herff_2014_nb_summary.png]

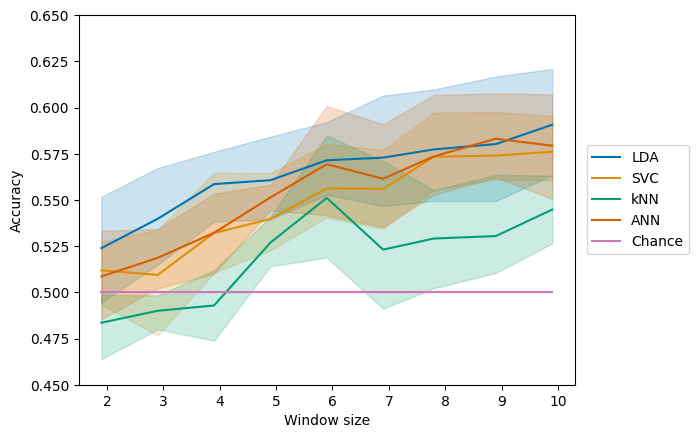

Supplement: Supplementary file 2 [file Data_Sheet_2.zip › benchnirs-v1.0/results/window_size/shin_2016_ma_summary.png]

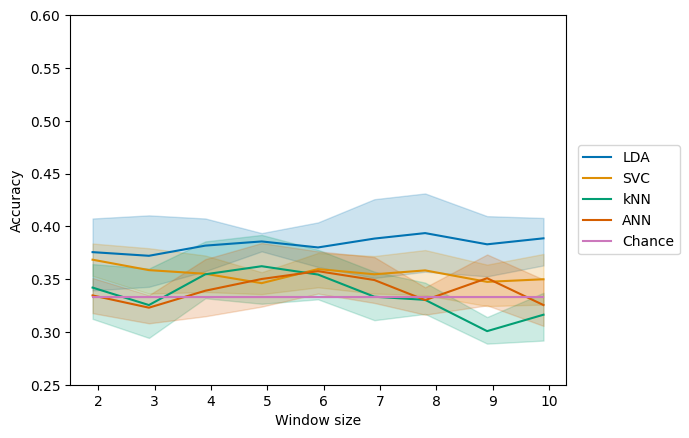

Supplement: Supplementary file 2 [file Data_Sheet_2.zip › benchnirs-v1.0/results/window_size/shin_2018_nb_summary.png]

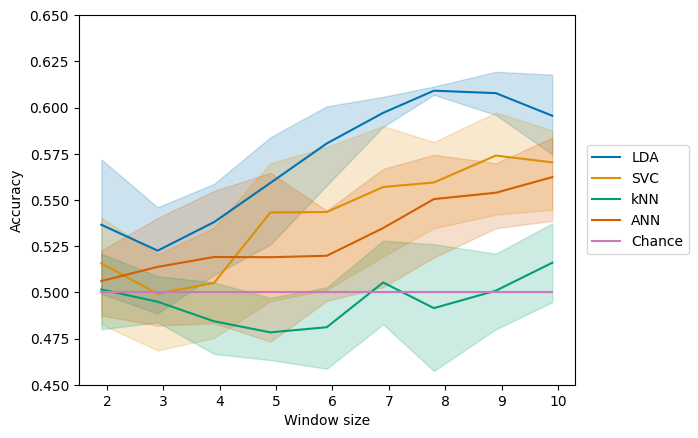

Supplement: Supplementary file 2 [file Data_Sheet_2.zip › benchnirs-v1.0/results/window_size/shin_2018_wg_summary.png]
